# Supplementary material for: Identifying key signs of motor neurone disease in primary care: a nested case–control study using the QResearch database
Source: BMJ Open. 2022 Jun 27;12(6):e058383. doi: 10.1136/bmjopen-2021-058383 (PMC9240876; doi:10.1136/bmjopen-2021-058383)
Supplement: Author's manuscript [file bmjopen-2021-058383.draft_revisions.pdf]

BMJ Open is committed to open peer review. As part of this commitment we make the peer review history of every article we publish publicly available.

When an article is published we post the peer reviewers' comments and the authors' responses online. We also post the versions of the paper that were used during peer review. These are the versions that the peer review comments apply to.

The versions of the paper that follow are the versions that were submitted during the peer review process. They are not the versions of record or the final published versions. They should not be cited or distributed as the published version of this manuscript.

BMJ Open is an open access journal and the full, final, typeset and author-corrected version of record of the manuscript is available on our site with no access controls, subscription charges or pay-per-view fees (http://bmjopen.bmj.com).

If you have any questions on BMJ Open's open peer review process please email info.bmjopen@bmj.com

BMJ Open

Identifying Key Signs of Motor Neurone Disease in Primary Care: A Nested Case-Control Study Using the QResearch Database

Journal:	*BMJ Open*
Manuscript ID	bmjopen-2021-058383
Article Type:	Original research
Date Submitted by the Author:	16-Oct-2021
Complete List of Authors:	Mei, Xue; Oxford University, Nuffield Department of Primary Care Health Sciences<br>Burchardt, Judith; Oxford University, Primary Care Health Sciences<br>Ranger, Tom; Oxford University, Primary Care Health Sciences<br>McDermott, Christopher; The University of Sheffield<br>Radunovic, Aleksandar; Barts Health NHS Trust<br>Coupland, Carol; University of Oxford, Nuffield Department of Primary Care Health Sciences; University of Nottingham<br>Hippisley-Cox, Julia; University of Oxford, Nuffield Department of Primary Care Health Sciences
Keywords:	Motor neurone disease < NEUROLOGY, EPIDEMIOLOGY, PRIMARY CARE

SCHOLARONE™
Manuscripts

I, the Submitting Author has the right to grant and does grant on behalf of all authors of the Work (as defined in the below author licence), an exclusive licence and/or a non-exclusive licence for contributions from authors who are: i) UK Crown employees; ii) where BMJ has agreed a CC-BY licence shall apply, and/or iii) in accordance with the terms applicable for US Federal Government officers or employees acting as part of their official duties; on a worldwide, perpetual, irrevocable, royalty-free basis to BMJ Publishing Group Ltd ("BMJ") its licensees and where the relevant Journal is co-owned by BMJ to the co-owners of the Journal, to publish the Work in this journal and any other BMJ products and to exploit all rights, as set out in our licence.

The Submitting Author accepts and understands that any supply made under these terms is made by BMJ to the Submitting Author unless you are acting as an employee on behalf of your employer or a postgraduate student of an affiliated institution which is paying any applicable article publishing charge ("APC") for Open Access articles. Where the Submitting Author wishes to make the Work available on an Open Access basis (and intends to pay the relevant APC), the terms of reuse of such Open Access shall be governed by a Creative Commons licence – details of these licences and which Creative Commons licence will apply to this Work are set out in our licence referred to above.

Other than as permitted in any relevant BMJ Author's Self Archiving Policies, I confirm this Work has not been accepted for publication elsewhere, is not being considered for publication elsewhere and does not duplicate material already published. I confirm all authors consent to publication of this Work and authorise the granting of this licence.

For peer review only

TITLE

Identifying Key Signs of Motor Neurone Disease in Primary Care: A Nested Case-Control Study Using the QResearch Database

Xue W Mei[1], Judith Burchardt[1], Tom A Ranger[1], Christopher J McDermott[2], Aleksandar Radunovic[3], Carol Coupland[1,4], Julia Hippisley-Cox[1]

[1]Nuffield Department of Primary Care Health Sciences, Woodstock Road, University of Oxford

[2]Sheffield Institute for Translational Neuroscience, University of Sheffield, UK

[3]Barts MND Centre, Barts Health NHS Trust, UK

[4] School of Medicine, University of Nottingham, UK

Correspondence to: Professor Julia Hippisley-Cox, Nuffield Department of Primary Care Health Sciences, Radcliffe Observatory Quarter, Woodstock Road, Oxford julia.hippisley-cox@phc.ox.ac.uk @juliahcox

Word count: 2,805 excluding references

For peer review only

For peer review only - http://bmjopen.bmj.com/site/about/guidelines.xhtml

ABSTRACT

Objective To identify key signs and symptoms in primary care electronic health records to improve early recognition, referral and diagnosis of motor neurone disease (MND).

Design A nested case-control study.

Setting 1292 UK general practices contributing to the QResearch primary care database, linked to hospital and mortality data.

Participants Baseline cohort included 16.8 million individuals aged 18 years and over without a diagnosis of MND at study entry and with more than 3 years of digitalised information available. The nested case-control dataset comprised of 6,437 cases of MND diagnosed between January 1998 and December 2019, matched by year of birth, gender, general practice, and calendar year to 62,003 controls.

Main outcome measures Clinically recognised symptoms and signs of MND prior to diagnosis as well as symptoms and factors which are relevant in primary care setting.

Results This study identified 17 signs and symptoms that were independently associated with MND diagnosis in a multivariable analysis. Of these, 10 were in the original Red Flag tool and 7 were new to the Red Flag tool (ataxia, dysphasia, weight-loss, wheeze, hoarseness of voice, urinary incontinence, and constipation). Amongst those from the Red Flag tool, dysarthria had the strongest association with subsequent MND (adjusted odds ratio (AOR): 43.5(95% CI 36.2 to 52.3)) followed by muscle fasciculations (AOR: 40.2 (95% CI 25.6 to 63.1)) and muscle wasting (AOR: 30.6 (95% CI 19.2 to 48.7)). Additionally, the associations between MND diagnosis and family history, dropped foot, focal weakness, and sialorrhoea remained robust after controlling for confounders such as body mass index and ethnicity, as well as other co-occurring symptoms. Patients that reported symptoms indicative of damage to the lower brainstem and its connections were diagnosed sooner than those who presented with respiratory or cognitive signs.

Conclusion This is the first study that has identified, validated and quantified the association of key symptoms and signs with MND diagnosis. Additional to known factors, the study has identified the following new factors to be independently associated with MND prior to diagnosis: ataxia, dysphasia, wheeze, and hoarseness of voice. These findings may be used to improve risk stratification and earlier detection of MND in primary care.

(360 words)

For peer review only - http://bmjopen.bmj.com/site/about/guidelines.xhtml

SUMMARY BOX

What is already known

Motor Neurone Disease (MND) is difficult to recognise in primary care and leads to diagnostic delay. Patients' quality of life can be greatly improved through early recognition and management of MND.

What this study add

Our study is the first to demonstrate that records in primary care can be used reliably to identify possible signs and symptoms to enable early recognition of MND. This is the first validation of the MND 'red flags' tool and has additionally identified new clinical features not conventionally attributed to MND which may serve as indicators for GPs when considering specialist referral.

Strengths and limitations

This study is to date, the largest nested-case control study derived from a representative population-based cohort looking at signs and symptoms related to MND.

We were able to collate an extensive list of signs and symptoms from the GP records, and to examine the association of each sign and symptom to subsequent MND diagnosis.

An important limitation to our study is the completeness and accuracy of the recording of the signs and symptoms, since not all patients with symptoms will seek medical attention and not all symptoms will be recorded at the GP consultations.

INTRODUCTION

Motor Neurone Disease (MND) causes progressive neuromuscular weakness that may first present as isolated and unexplained symptoms. MND causes the deaths of 1 in 350 men and 1 in 470 women in the UK[1], although there is emerging evidence that the incidence might be higher than previously thought[2]. MND is difficult to recognise in primary care since it is both a relatively uncommon disease and the clinical presentations of early symptoms are sporadic and non-specific. As a result, patients may delay consulting their general practitioners (GPs) and the GPs may not attribute the symptoms to MND. Previous work has demonstrated that both patient factors (e.g. delayed presentation to GP) and health care practice factors (e.g. referrals to non-neurology specialists) contribute to delays in MND diagnosis hence patients' access to disease management[3].

In 2014, the Motor Neurone Disease Association and the Royal College of General Practitioners (RCGP) collaborated to produce a Red Flag tool for MND[4], which is designed to improve timely referrals to neurology specialists from primary care consultants. This tool was developed based on interviews with MND specialists and details a number of symptoms and signs which could be indicative of MND but has not been verified by analysis of clinical data. Therefore the aims of our study are to validate the symptoms and signs in the Red Flag tool; to quantify the extent to which the key symptoms and signs are associated with MND; as well as to identify additional factors which may be helpful within the primary care setting in recognition of possible MND and triggering timely referral to neurology specialists.

METHOD

Study design

For peer review only - http://bmjopen.bmj.com/site/about/guidelines.xhtml

We conducted a nested case-control study in a large UK primary care cohort using the QResearch database (version 44). QResearch is an anonymised, large open cohort database which prospectively collects routine health data from general practices in the UK that use the Egton Medical Information Systems (EMIS) computer system (used by 55% of GPs in the UK). The data include individual level data for demographics, lifestyle factors, medical diagnoses, prescriptions, specialist referrals, medical examinations, and examination results and are linked to Hospital Episode Statistics (HES) records and Office of National Statistics (ONS) death registry records.

Ascertainment of cases and controls

The base cohort included individuals aged 18 years and over registered during the study period (1 January 1998 to 31 December 2019) without a diagnosis of MND at study entry. The study entry date was defined as the latest of any of the following: 18th birthday; date of registration with the practice plus one year; date on which the practice computer system was installed plus one year; or the beginning of the study period (1 January 1998). The cohort was followed up until the earliest of any of the following: the date of MND diagnosis; date of 100th birthday; date of death; date of leaving the practice; or the study end date (31 December 2019).

Cases were defined as patients in the study cohort with a new diagnosis of MND on any of the GP record, Hospital Episode Statistics (HES) record or Office of National Statistics (ONS) death registry during follow-up. The following Read codes were used to identify the cases from the GP records: F152 (motor neurone disease), F1520 (amyotrophic lateral sclerosis (ALS)), F1521 (progressive muscular atrophy), F1522 (progressive bulbar palsy), F1523 (pseudobulbar palsy), F1524 (primary lateral sclerosis), and F152z (MND not otherwise specified). We also used ICD10 code G122 (motor neurone disease) to identify further records from HES and ONS death registry.

Controls were matched to cases by sex, practice, year of birth, and calendar year using the incidence density sampling method[5]. Each control was allocated an index date which was the date of first MND diagnosis of their matched cases. Each case was matched with up to 10 controls who were alive, registered, and without a diagnosis of MND at the index date. Cases and controls were excluded if they had less than three years of computerised data available at the index date, to ensure that the data are complete for a minimum of three years prior to index date.

Symptoms

Red flag symptoms included clinically recognised symptoms and signs of MND[4 6]. The list of existing factors are as following: bulbar features - dysarthria, dysphagia, sialorrhoea or excessive salivation, and tongue fasciculations; limb or muscle features - focal weakness, falls, foot drop, muscle wasting, muscle fasciculations or twitching, cramps, sensory impairment, and muscle stiffness; respiratory features - orthopnoea, dyspnoea, shortness of breath on exertion, sleep problems, tiredness or fatigue, and early morning headache; cognitive features – behavioural change, emotional lability, depression, hallucinations, and confusion; as well as appetite loss and family history of MND as supporting factors. Additionally, we included potential symptoms such as weight-loss, ataxia, dry mouth, heaviness of legs, and difficulty climbing stairs which were identified through literature searches[7-9] and a search of Read/SNOMED-CT codes for symptoms recorded prior to diagnosis in a pilot study involving a sample of patients diagnosed with MND in JB's general practice. These were included because although not directly established signs/symptoms of MND, these may provide valuable information in identifying additional patients who may benefit from neurologist referral in the primary care setting. We also included the following list of conditions to serve as negative controls since these are not usually considered to be associated with MND: diplopia, ptosis, bladder and bowel

For peer review only - http://bmjopen.bmj.com/site/about/guidelines.xhtml

problems such as incontinence and constipation, as well as sexual dysfunction. All symptoms and signs were identified through searches of the GP records via corresponding Read/SNOMED-CT codes. For the primary analyses, only signs and symptoms that were last recorded between 3 months to 5 years prior to index date were included, as symptoms occurring more than 5 years ago were considered unlikely to be relevant to MND and those recorded less than 3 months prior are likely to be reflective of the average waiting period between specialist referral and formal diagnosis. We also excluded very rare signs and symptoms (defined as < 5 ever recorded counts in either cases or controls). Table 2 has a summary of the factors included in the analysis and the source of the information.

Statistical analysis

We estimated the strength of association of the red flag signs and symptoms with MND diagnosis, expressed as odds ratios (ORs), using conditional logistic regression. We performed both univariate and multivariable analysis, adjusted for the following confounders: the most recently recorded body mass index (BMI) prior to index date, latest records of smoking status and alcohol consumption, Townsend deprivation score in quintiles, and self-assigned ethnic groups. We assumed missing data on BMI, smoking, alcohol consumption, Townsend scores, and ethnic groups were missing at random and imputed them using multiple imputation by chained equations[10-12]. We created 20 imputed datasets and the imputation model included demographic variables, comorbidities or disease history (I.e. ever diagnosis of one or more of the following: cardiovascular diseases, stroke, diabetes (type 1 and 2), dementia, Parkinson's disease, myasthenia gravis, and multiple sclerosis), all symptoms and signs that were included in the final regression model, as well as the case-control indicator. The ORs from each imputed dataset were combined to form the final adjusted ORs using Rubin's rule[13]. Only signs and symptoms that reached statistical significance (two-tailed $P < 0.05$) in the univariate analysis were included in the multivariate analysis.

Patient and public involvement

This study was initiated by the Motor Neurone Disease Association which is a charity representing patients with MND. Apart from this, patient and public were not involved in design and development of the study or interpretation of the study outcome but will be consulted regarding the dissemination of the results.

RESULTS

Descriptive characteristics of the study cohort

The base cohort comprised of 16,799,992 participants aged between 18 and 100 years. During a total of 112,003,453 person-years of follow-up, we found 6,437 incident cases of MND that were eligible for inclusion, using records from general practice, hospital episode statistics, and death registry databases. We were able to identify a total of 62,003 birth year, gender, practice, and calendar year matched controls. 56.8% of the MND cases were men and mean (SD) age at diagnosis was 69.6 (12.2) years.

Table 1 is a summary of the demographic characteristics of the cases and controls. The ethnicity recording was more complete in controls (71.3%) than in cases (55.4%), however, completeness of Townsend deprivation score, alcohol, smoking, and BMI were similar between the two groups. There was no significant difference between cases and controls in terms of alcohol consumption or smoking status. However, compared with the controls, the cases had lower BMI ($P < 0.001$) and were more likely to be living in less deprived areas based on Townsend score quintiles ($P = 0.013$).

For peer review only - http://bmjopen.bmj.com/site/about/guidelines.xhtml

Table 1. Demographic characteristics of case patients with motor neurone disease and age, sex practice, calendar time matched controls. Figures are numbers (%) unless otherwise indicated.

Characteristic	Study Participants, No. (column %)	
	controls (n = 62,003)	cases (n= 6,437)
Women	27058(43.6)	2781(43.2)
Men	34945(56.4)	3656(56.8)
Age at diagnosis/index date (mean(SD)), y	69.1(11.9)	69.6(12.2)
Age at study entry		
< 50	10700(17.3)	1078(16.7)
50 - 54	6556(10.6)	651(10.1)
55 - 59	8016(12.9)	793(12.3)
60 - 64	9678(15.6)	976(15.2)
65 - 69	9734(15.7)	982(15.3)
70 - 74	8085(13)	852(13.2)
75 - 79	5771(9.3)	627(9.7)
80 +	3463(5.6)	478(7.4)
Ethnicity recorded	44211 (71.3)	3563 (55.4)
**Ethnicity**		
White	41106(66.3)	3281(51)
Indian	700(1.1)	69(1.1)
Pakistani	349(0.6)	32(0.5)
Bangladeshi	174(0.3)	19(0.3)
Other Asian	322(0.5)	30(0.5)
Caribbean	537(0.9)	48(0.7)
Black African	373(0.6)	28(0.4)
Chinese	132(0.2)	4(0.1)
Other	518(0.8)	52(0.8)
Not recorded	17792(28.7)	2874(44.6)
**Townsend deprivation score, quintiles**		
1 (least deprived)	20410(32.9)	2205(34.3)
2	16131(26)	1613(25.1)
3	11533(18.6)	1174(18.2)
4	8263(13.3)	795(12.4)
5 (most deprived)	5587(9)	640(9.9)
Townsend deprivation score not recorded	79(0.1)	10(0.2)
BMI not recorded before index date	11352(18.3)	1124 (17.5)
BMI (mean(SD))	26.6(4.6)	26.2(4.5)
**Smoking status**		
Non-smoker	29655(47.8)	3142(48.8)
Ex-smoker	17756(28.6)	1882(29.2)
Light smoker (1-9 cigarettes/day)	5931(9.6)	596(9.3)
Moderate smoker (10-19 cigarettes/day)	1125(1.8)	109(1.7)

For peer review only

For peer review only - http://bmjopen.bmj.com/site/about/guidelines.xhtml

Characteristic	Study Participants, No. (column %)	
	controls (n = 62,003)	cases (n= 6,437)
Heavy smoker (≥20 cigarettes/day)	816(1.3)	76(1.2)
Smoking not recorded before index date	6720(10.8)	632(9.8)
**Alcohol consumption**		
Non-drinker	31677(51.1)	3454(53.7)
Trivial (<1U/day)	9215(14.9)	906(14.1)
Light (1-2u/day)	4460(7.2)	465(7.2)
Moderate (3-6u/day)	4582(7.4)	453(7)
Heavy (7-9u/day)	343(0.6)	30(0.5)
Very Heavy (>9u/day)	129(0.2)	15(0.2)
Alcohol not recorded before index date	11597(18.7)	1114(17.3)

Association of red flag signs and symptoms to MND diagnosis

The types of symptoms and signs as well as their adjusted estimates are presented in Figure 1 (univariate) and Figure 2 (multivariate). Table 2 also contains the list of factors examined, source from which they were obtained, and whether they were statistically significant in the analysis. 33 signs and symptoms reached our pre-specified level of statistical significance (two-tailed $P < 0.05$) in the unadjusted univariate analysis, and these remained significant after adjustment for BMI, alcohol consumption, smoking, deprivation, and self-reported ethnicity (Figure 1). These were collectively included in a multivariable model for estimation of their independent associations with MND diagnosis. Dysarthria was the strongest independent predictor of MND diagnosis (OR 43.2, 95%CI 36.0-52.0) followed by muscle fasciculations (OR (95%CI) 40.2 (25.6-63.1)), muscle wasting (OR (95%CI) 31.0 (19.5-49.4)), and dropped foot (OR (95%CI) 14.8 (11.3-19.3)). Some respiratory symptoms such as shortness of breath and dyspnoea, as well as cognitive symptoms such as depression and confusion, though statistically significant in univariate analysis, were no longer associated with MND after controlling for other symptoms and signs. We also found the following symptoms, which were not included in the RF tool but were found in GP records, to be independently associated with MND diagnosis: hoarseness of voice (OR (95%CI) 3.26 (2.82-3.77)), dysphasia (OR (95%CI) 2.85 (1.92-4.23)), ataxia (OR (95%CI) 4.82 (3.58-6.48)), wheeze (OR (95%CI) 1.15 (1.01-1.32)), and weight-loss (OR (95%CI) 2.20 (1.92-2.52)).

Table 2 Factors included for analysis and the source from which they were identified[1]

	**Symptoms and signs**	**Source/Reason for inclusion**	**Confirmed on analysis[2]**
**Bulbar features**			
	Dysarthria	MNDA	UV, MV
	Dysphagia	MNDA	UV, MV
	Sialorrhoea/excessive salivation	MNDA	UV, MV
	Tongue fasciculations	MNDA	UV
**Limb Features/muscle weakness**			
	Focal weakness	MNDA	UV, MV

For peer review only - http://bmjopen.bmj.com/site/about/guidelines.xhtml

	Symptoms and signs	Source/Reason for inclusion	Confirmed on analysis[2]
**Bulbar features**			
	Falls	MNDA	UV, MV
	Foot drop	MNDA	UV, MV
	Muscle wasting	MNDA	UV, MV
	Muscle fasciculations or twitching	MNDA	UV, MV
	Cramps	MNDA	UV, MV
	Sensory impairment	MNDA	No
	Muscle stiffness	NICE	UV, MV
**Respiratory features**			
	Orthopnoea	MNDA	No
	Dyspnoea	MNDA	UV
	Shortness of breath on exertion	MNDA	UV
	Sleep problems	MNDA	UV
	Tiredness/fatigue	MNDA	UV
	Early morning headache	MNDA	No
**Cognitive features**			
	Behavioural change	MNDA	No
	Emotional lability	MNDA	UV
	Depression	MNDA	UV
	Hallucinations	NICE	UV
	Confusion	NICE	UV
**Supporting factors**			
	Family history of MND	MNDA	UV, MV
	Appetite loss	NICE	UV
**Additional factors from literature review/pilot study**			
	Alcoholism	Literature	No
	Weight-loss	Literature	UV, MV
	Ataxia	Pilot study	UV, MV
	Wheeze	Pilot study	UV, MV
	Difficulty climbing stairs	Pilot study	UV
	Dry mouth	Pilot study	UV
	Dysphasia	Pilot study	UV, MV

For peer review only

For peer review only - http://bmjopen.bmj.com/site/about/guidelines.xhtml

	Symptoms and signs	Source/Reason for inclusion	Confirmed on analysis[2]
**Bulbar features**			
	Hoarseness of voice	Pilot study	UV, MV
**Factors not considered supportive of diagnosis by MNDA**			
	Urinary frequency increase	Negative control	UV
	Urinary incontinence	Negative control	UV, MV
	Urinary retention	Negative control	UV
	Nocturia	Negative control	UV
	Dysuria	Negative control	No
	Constipation	Negative control	UV, MV
	Faecal incontinence	Negative control	No
	Impotence	Negative control	No
	Diplopia	Negative control	No
	Ptosis	Negative control	No

[1] The study was matched on age and gender therefore they were not included in the analysis model

[2] Indicates the analysis model used to find the associations. All models were additionally adjusted for Body Mass Index, Townsend scores in quintiles, smoking status, alcohol consumption behaviour, and self-reported ethnicity; UV Univariate, MV Multivariate

(Figure 1)

We also observed independent associations with MND for two of the symptoms not generally considered to be associated with MND: constipation (OR (95%CI) 1.45 (1.31-1.60)) and urinary incontinence (OR (95%CI) = 1.28 (1.12-1.47)).

Overall, symptoms from bulbar dysfunction (i.e. dysarthria, dysphagia, sialorrhoea, and tongue fasciculations) and those appearing in the limbs are most strongly suggestive of subsequent MND diagnosis. Symptoms and signs occurring in the respiratory and cognitive systems were not statistically significant when evaluated in combination with others.

(Figure 2)

Time from earliest symptom presentation to MND diagnosis

Duration from the earliest symptom presentation to MND diagnosis varied considerably by feature and location of the signs and symptoms. Patients presenting with symptoms and signs associated with damage of bulbar region and limb functions received their diagnoses sooner than those presenting with respiratory or cognitive symptoms and signs (e.g. median days from symptom presentation to diagnosis: dysarthria 145.5 days (IQR 68.5 – 296 days) vs. orthopnoea 299 days (IQR 42 – 496 days)). Amongst those clinical features, unusual and sporadic signs such as dysarthria and muscle

For peer review only

For peer review only - http://bmjopen.bmj.com/site/about/guidelines.xhtml

fasciculations appeared to be the key indicators to speedy neurologist referral. Time from earliest clinical symptom presentation to MND diagnosis (median days, IQR) is presented in Figure 3.

(Figure 3)

DISCUSSION

Principal findings

In this very large population based nested case-control study covering a population of over 15 million adults, we were able to demonstrate that the Motor Neurone Disease Association recommended Red Flag signs and symptoms[4], particularly those affecting bulbar and limb regions, are independently predictive of subsequent MND diagnosis. Amongst our study of 6,437 MND patients and 62,003 matched controls, the symptom with the largest association with MND was dysarthria followed by muscle fasciculations and muscle wasting. Additionally from the GP records, we found the following symptoms to be independently associated with MND: hoarseness of voice, dysphasia, ataxia, wheeze, and weight-loss. We also found a higher proportion of MND cases with documented constipation and urinary incontinence than the controls.

Consistent with previous studies[14 15], we found patients showing bulbar onset symptoms such as dysarthria and dysphagia were diagnosed on average 4-5 months sooner than those with limb onset symptoms. Interestingly in our cohort, patients presenting with muscle fasciculations were diagnosed most quickly, which is likely as a result of timely and adequate specialist referral by their managing GPs. This is consistent with a qualitative study conducted by Baxter and McDermott[16] which explored the decision-making and referral process of GPs through semi-structured interviews. The study found that among the 42 GPs who consented to participate, fasciculation was the most commonly described trigger for suspected MND and neurologist referral[16]. Our finding demonstrated that muscle fasciculation is indeed likely one of the top triggers for specialist referral practised by GPs across England. We found the patients who exhibited speech related symptoms such as dysarthria received a diagnosis within an average of 6 months of symptom presentation. This suggests that the awareness of speech impairment in neurological disorders is relatively high amongst GPs in England, in contrast to previous studies[16].

Signs and symptoms of respiratory and cognitive systems, while moderately associated with MND in univariate analysis, were no longer significant after accounting for symptoms related to bulbar region impairment and limb functions. The period between initial presentation of respiratory and cognitive symptoms and MND diagnosis is also significantly longer. This finding seems to indicate that presentations of respiratory or cognitive featured symptoms alone were insufficient to trigger a specialist referral. One reason for this may be that the symptom onset is relatively mild and sporadic, and may not alarm the managing GPs since many other patients also have comorbidities with similar symptom profile. However, GPs could cross reference these symptoms against others (i.e. dysarthria or muscle wasting) to enable neurologist referral.

The positive associations between constipation and urinary incontinence with MND found in our study were unexpected since these have not been described in MND before[8 17]. We suspect the observed effect is primarily due to reverse causality: patients with undiagnosed but advanced MND are likely to have trouble with balance and movement, thereby hindering their ability to perform tasks such as going to the toilet.

For peer review only - http://bmjopen.bmj.com/site/about/guidelines.xhtml

Strengths and limitations

This study is to date, the largest nested-case control study derived from a representative population-based cohort looking at signs and symptoms related to MND. We were able to collate an extensive list of signs and symptoms from the GP records, and to examine the association of each sign and symptom to subsequent MND diagnosis. We were also able to demonstrate that duration of the earliest symptom presentation to MND diagnosis could serve as an indicator to key signs and symptoms that triggered neurologist referral. We could not determine the time point, number, and types of specialist referrals because our study was designed to compare the pattern of symptoms prior to diagnosis in cases (and the equivalent index date for controls). Another important limitation to our study is the completeness and accuracy of the recording of the signs and symptoms, since not all patients with symptoms will seek medical attention and not all symptoms will be recorded at the GP consultations. Furthermore, the positive associations may be attributed to unmeasured confounding or variation in coding practices at the GPs, due to the nature of routinely collected data. For example, we found dysphasia and wheeze to be independently and positively associated with MND. However these symptoms are not traditionally considered within the clinical features of MND although dysphasia has been described to present in ALS in a systematic review[18]. It might also be that the patients presented with both dysarthria (disorder of speech) and dysphasia (disorder of language) but only dysphasia was coded because it was the dominant symptom when GP consultation was sought. Similarly, shortness of breath on exertion is a well-recognised sign of MND and might be coded as wheeze if symptom presentation of respiratory function tests are not undertaken.

CONCLUSION

In this very large population based nested case-control study of 6,437 MND cases and 62,003 matched controls, we were able to validate the currently recognised signs and symptoms, particularly those of bulbar and limb regions to be independently associated with MND. Additionally, we found GP recorded hoarseness of voice, dysphasia, ataxia, and weight-loss to be associated with subsequent MND diagnosis. Due to the nature of the data as highlighted in the limitations, we could not verify whether all positive findings were true symptoms caused by MND or whether they were due to patient presentation patterns or GP coding behaviour. However, these findings have implications for GPs when considering referrals to neurologists and may be used to improve risk stratification and prediction of MND in primary care.

Future studies may focus on chronologically mapping the symptoms, characterising the patterns and clusters in symptoms presentation, and predicting disease progression based on types and duration of existing symptoms.

CONTRIBUTORS

XM undertook data manipulation, reviewed the literature, led the data analysis, undertook interpretation of the data, and wrote the first draft of the paper. JHC led the study, obtained funding, data approvals, designed the study and drafted the protocol, contributed to the data management and interpretation of the data. CJM contributed to the funding application, contributed to protocol development and interpretation of results. CC contributed to the funding application, the discussion on protocol development and provided critical feedback on drafts of the manuscript. JB reviewed the literature on presentation of MND and conducted a search of how it had presented in patients in her own general practice. TR contributed to interpretation of findings and compilation of the manuscript. All authors approved the protocol, contributed to the critical revision of the manuscript

For peer review only - http://bmjopen.bmj.com/site/about/guidelines.xhtml

and approved the final version of the manuscript. The corresponding author attests that all listed authors meet authorship criteria and that no others meeting the criteria have been omitted.

FUNDING

This study was funded by a grant from Motor Neurone Disease Association (Hippisley-Cox/Nov18/939-794). The funders of this study contributed to the design and conduct of the study and reviewed and approve the manuscript. The funders had no role in analysis or interpretation of data. XM, TR, CC, JHC had full access to all the study data and JHC had final responsibility for submission.

COMPETING INTEREST

All authors have completed the ICMJE uniform disclosure form at www.icmje.org/coi_disclosure.pdf. JHC reports grants from grants from the John Fell Oxford University Press Research Fund; Cancer Research UK (CR-UK) grant number C5255/A18085, through the Cancer Research UK Oxford Centre and the Oxford Wellcome Institutional Strategic Support Fund (204826/Z/16/Z), during the conduct of the study. JHC is an unpaid director of QResearch, a not-for-profit organisation which is a partnership between the University of Oxford and EMIS Health who supply the QResearch database used for this work. JHC is a founder and shareholder of ClinRisk ltd and was its medical director until 31st May 2019. JHC is member of the SAGE subgroups on ethnicity and data and is chair of the risk stratification subgroup of NERVTAG. The views expressed are those of the authors only. CJM is supported by the NIHR Sheffield Biomedical Centre.

DATA SHARING

To guarantee the confidentiality of personal and health information only the authors have had access to the data during the study in accordance with the relevant licence agreements. Access to the QResearch data is according to the information on the QResearch website (www.qresearch.org).

TRANSPARENCY

XM, TR, and JHC had full access to all data in the study and takes responsibility for the integrity of the data and the accuracy of the data analysis. JHC is the guarantor for the study and affirms that the manuscript is an honest, accurate, and transparent account of the study reported; that no important aspects of the study have been omitted; and that any discrepancies from the study as planned have been explained.

DISSEMINATION TO PARTICIPANTS AND RELATED PATIENT AND PUBLIC COMMUNITIES

We will work with the Motor Neurone Disease association to ensure the results are disseminated to the patient and public communities

PROVENANCE AND PEER REVIEW

Not commissioned; externally peer reviewed.

For peer review only - http://bmjopen.bmj.com/site/about/guidelines.xhtml

COPYRIGHT STATEMENT/LICENCE FOR PUBLICATION

"I, the Submitting Author has the right to grant and does grant on behalf of all authors of the Work (as defined in the author licence), an exclusive licence on a worldwide, perpetual, irrevocable, royalty-free basis to BMJ Publishing Group Ltd ("BMJ") its licensees. The Submitting Author accepts and understands that any supply made under these terms is made by BMJ to the Submitting Author unless I am acting as an employee on behalf of my employer which is paying any applicable article publishing charge ("APC") for Open Access articles. Where the Submitting Author wishes to make the Work available on an Open Access basis (and intends to pay the relevant APC), the terms of reuse of such Open Access shall be governed by a Creative Commons licence – details of these licences and which licence will apply to this Work are set out in our licence referred to above."

ETHICS STATEMENT

The QResearch® ethics approval is with East Midlands-Derby Research Ethics Committee [reference 18/EM/0400, project reference OX1].

For peer review only

REFERENCES

1. Alonso A, Logroscino G, Jick SS, et al. Incidence and lifetime risk of motor neuron disease in the United Kingdom: a population-based study. *Eur J Neurol* 2009;16(6):745-51.
2. Burchardt J, Mei X, Ranger T, et al. Analysis of Incidence of Motor Neuron Diseae in England 1998-2019: Use of Three Linked Datasets. *Manuscript submitted for publication* 2021
3. Richards D, Morren JA, Pioro EP. Time to diagnosis and factors affecting diagnostic delay in amyotrophic lateral sclerosis. *Journal of the Neurological Sciences* 2020;417:117054. doi: https://doi.org/10.1016/j.jns.2020.117054
4. Association MND. Red Flag Diagnosis Tool [Available from: https://www.mndassociation.org/app/uploads/2013/10/red-flags-final-2.pdf accessed July 2020.
5. Vandenbroucke JP, Pearce N. Case–control studies: basic concepts. *International Journal of Epidemiology* 2012;41(5):1480-89. doi: 10.1093/ije/dys147
6. (NICE) NIfHaCE. Motor neurone disease: assessment and management 2016 [Available from: https://www.nice.org.uk/guidance/ng422019.
7. Kiernan MC, Vucic S, Cheah BC, et al. Amyotrophic lateral sclerosis. *The Lancet* 2011;377(9769):942-55. doi: 10.1016/S0140-6736(10)61156-7
8. McDermott CJ, Shaw PJ. Diagnosis and management of motor neurone disease. *BMJ* 2008;336(7645):658. doi: 10.1136/bmj.39493.511759.BE
9. Nageshwaran S, Davies LM, Rafi I, et al. Motor neurone disease. *BMJ : British Medical Journal* 2014;349
10. Group TAM. Academic Medicine: problems and solutions. *British Medical Journal* 1989;298:573-79.
11. Schafer J, Graham J. Missing data: our view of the state of the art. *Psychological Methods* 2002;7:147-77.
12. Steyerberg EW, van Veen M. Imputation is beneficial for handling missing data in predictive models. *J Epidemiol Community Health* 2007;60:979.
13. Rubin DB. Multiple Imputation for Non-response in Surveys. New York: John Wiley 1987.
14. Galvin M, Ryan P, Maguire S, et al. The path to specialist multidisciplinary care in amyotrophic lateral sclerosis: A population- based study of consultations, interventions and costs. *PloS one* 2017;12(6):e0179796-e96. doi: 10.1371/journal.pone.0179796
15. Kraemer M, Buerger M, Berlit P. Diagnostic problems and delay of diagnosis in amyotrophic lateral sclerosis. *Clin Neurol Neurosurg* 2010;112(2):103-5. doi: 10.1016/j.clineuro.2009.10.014 [published Online First: 2009/11/26]
16. Baxter S, McDermott CJ. Decision-making and referral processes for patients with motor neurone disease: a qualitative study of GP experiences and evaluation of a new decision-support tool. *BMC Health Serv Res* 2017;17(1):339. doi: 10.1186/s12913-017-2286-0 [published Online First: 2017/05/10]
17. Foster LA, Salajegheh MK. Motor Neuron Disease: Pathophysiology, Diagnosis, and Management. *The American Journal of Medicine* 2019;132(1):32-37. doi: https://doi.org/10.1016/j.amjmed.2018.07.012
18. Pinto-Grau M, Hardiman O, Pender N. The Study of Language in the Amyotrophic Lateral Sclerosis - Frontotemporal Spectrum Disorder: a Systematic Review of Findings and New Perspectives. *Neuropsychology Review* 2018;28(2):251-68. doi: 10.1007/s11065-018-9375-7

For peer review only - http://bmjopen.bmj.com/site/about/guidelines.xhtml

Figure 1 Univariate analysis for risk of MND diagnosis based on 6,437 cases and 62,003 age, sex, practice matched controls

Figure 2 Multivariate analysis for risk of MND diagnosis based on 6,437 cases and 62,003 age, sex, practice matched controls

Figure 3 Median duration (days, IQR, from earliest symptom presentation to MND Diagnosis

For peer review only

For peer review only - http://bmjopen.bmj.com/site/about/guidelines.xhtml

Figure 1. Univariate analysis for risk of MND diagnosis based on 6,437 cases and 62,003 age, sex, practice matched controls

adjusted for variables shown, age, BMI, alcohol, smoking, ethnicity, deprivation

Symptoms	Cases	Controls	OR (95% CI)	Feature
Dysarthria**	362 (5.6)	124 (0.2)	36.51 (29.27, 45.54)	Bulbar
Sialorrhoea**	29 (0.5)	21 (< 0.1)	12.95 (7.29, 23.03)	Bulbar
Dysphagia**	295 (4.6)	544 (0.9)	6.50 (5.62, 7.52)	Bulbar
Tongue fasciculations**	35 (0.5)	181 (0.3)	1.83 (1.27, 2.65)	Bulbar
Muscle fasciculations**	65 (1)	15 (< 0.1)	50.90 (28.22, 91.80)	Limb
Muscle wasting**	53 (0.8)	21 (< 0.1)	25.69 (15.28, 43.19)	Limb
Dropped foot**	129 (2)	68 (0.1)	20.24 (14.97, 27.37)	Limb
Focal weakness**	364 (5.7)	351 (0.6)	12.19 (10.42, 14.26)	Limb
Falls**	846 (13.1)	3217 (5.2)	3.57 (3.26, 3.92)	Limb
Cramp**	318 (4.9)	1283 (2.1)	2.56 (2.25, 2.91)	Limb
Muscle stiffness*	39 (0.6)	170 (0.3)	2.41 (1.71, 3.41)	Limb
Sensory impairement**	120 (1.9)	1036 (1.7)	1.17 (0.97, 1.42)	Limb
Nocturia	146 (2.3)	841 (1.4)	1.67 (1.39, 2.00)	Respiratory
Shortness of breath**	443 (6.9)	3070 (5)	1.53 (1.37, 1.70)	Respiratory
Tiredness**	470 (7.3)	3251 (5.2)	1.48 (1.33, 1.64)	Respiratory
Dyspnoea**	550 (8.5)	3927 (6.3)	1.47 (1.33, 1.62)	Respiratory
Orthopnoea**	9 (0.1)	66 (0.1)	1.31 (0.65, 2.66)	Respiratory
Sleep problems**	443 (6.9)	3443 (5.6)	1.29 (1.16, 1.44)	Respiratory
Early morning headache**	338 (5.3)	3018 (4.9)	1.11 (0.99, 1.25)	Respiratory
Hallucinations*	13 (0.2)	43 (0.1)	2.55 (1.30, 5.00)	Cognitive
Emotional lability**	26 (0.4)	137 (0.2)	1.95 (1.28, 2.96)	Cognitive
Depression**	263 (4.1)	1521 (2.5)	1.81 (1.57, 2.07)	Cognitive
Confusion*	70 (1.1)	433 (0.7)	1.54 (1.19, 1.99)	Cognitive
Behavioural change**	14 (0.2)	79 (0.1)	1.53 (0.86, 2.74)	Cognitive
Family history of MND**	19 (0.3)	10 (< 0.1)	19.04 (8.80, 41.18)	Supporting factor
Appetite loss*	55 (0.9)	279 (0.4)	1.94 (1.44, 2.61)	Supporting factor
Weightloss	254 (3.9)	910 (1.5)	2.93 (2.53, 3.39)	Literature
Alcoholism	43 (0.7)	357 (0.6)	1.21 (0.88, 1.67)	Literature
Dysphasia	48 (0.7)	52 (0.1)	9.78 (6.49, 14.74)	Pilot study
Ataxia	71 (1.1)	95 (0.2)	7.83 (5.73, 10.71)	Pilot study
Hoarseness of voice	274 (4.3)	518 (0.8)	5.47 (4.69, 6.38)	Pilot study
Difficulty climbing stairs	17 (0.3)	81 (0.1)	2.24 (1.25, 4.00)	Pilot study
Dry mouth	100 (1.6)	575 (0.9)	1.68 (1.35, 2.09)	Pilot study
Wheeze	609 (9.5)	4459 (7.2)	1.46 (1.33, 1.61)	Pilot study
Constipation	628 (9.8)	3119 (5)	2.17 (1.97, 2.38)	Not supporting factor
Urinary incontinence	244 (3.8)	1237 (2)	2.01 (1.74, 2.33)	Not supporting factor
Urinary retention	84 (1.3)	508 (0.8)	1.56 (1.23, 1.98)	Not supporting factor
Ptosis	17 (0.3)	118 (0.2)	1.55 (0.95, 2.53)	Not supporting factor
Faecal incontinence	11 (0.2)	65 (0.1)	1.52 (0.79, 2.91)	Not supporting factor
Diplopia	30 (0.5)	201 (0.3)	1.46 (0.99, 2.14)	Not supporting factor
Urinary frequency increase	214 (3.3)	1515 (2.4)	1.38 (1.19, 1.61)	Not supporting factor
Impotence	251 (3.9)	2378 (3.8)	1.04 (0.91, 1.20)	Not supporting factor
Dysuria	103 (1.6)	984 (1.6)	0.98 (0.80, 1.21)	Not supporting factor

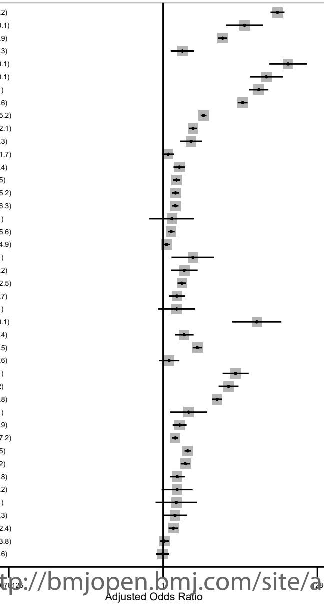

Model included signs and symptoms that were last recorded between 3 months to 5 years prior to MND diagnosis or index date

QResearch database version 44, 2020, University of Oxford, Mei et al

For peer review only - http://bmjopen.bmj.com/site/about/guidelines.xhtml

Figure 2. Multivariate analysis for risk of MND diagnosis based on 6,437 cases and 62,003 age, sex, practice matched controls

adjusted for variables shown, age, BMI, alcohol, smoking, ethnicity, deprivation

Symptoms	Cases	Controls	OR (95% CI)	Feature
Dysarthria**	790 (12.3)	214 (0.3)	43.22 (35.95, 51.97)	Bulbar
Sialorrhoea**	54 (0.8)	35 (0.1)	7.96 (4.50, 14.08)	Bulbar
Dysphagia**	652 (10.1)	1110 (1.8)	4.57 (4.01, 5.21)	Bulbar
Tongue fasciculations**	76 (1.2)	363 (0.6)	1.29 (0.93, 1.78)	Bulbar
Muscle fasciculations**	134 (2.1)	34 (0.1)	40.19 (25.59, 63.13)	Limb
Muscle wasting**	102 (1.6)	39 (0.1)	31.02 (19.46, 49.44)	Limb
Dropped foot**	208 (3.2)	126 (0.2)	14.78 (11.33, 19.28)	Limb
Focal weakness**	736 (11.4)	599 (1)	14.00 (12.20, 16.07)	Limb
Falls**	1304 (20.3)	5290 (8.5)	2.70 (2.46, 2.97)	Limb
Cramp**	470 (7.3)	2427 (3.9)	1.54 (1.35, 1.76)	Limb
Muscle stiffness*	84 (1.3)	446 (0.7)	1.53 (1.15, 2.03)	Limb
Tiredness**	903 (14)	6545 (10.6)	1.08 (0.98, 1.20)	Respiratory
Shortness of breath**	728 (11.3)	5031 (8.1)	1.00 (0.84, 1.18)	Respiratory
Dyspnoea**	819 (12.7)	5990 (9.7)	0.96 (0.84, 1.10)	Respiratory
Sleep problems**	789 (12.3)	6151 (9.9)	0.92 (0.83, 1.02)	Respiratory
Hallucinations*	22 (0.3)	74 (0.1)	1.21 (0.62, 2.35)	Cognitive
Depression**	545 (8.5)	4001 (6.5)	1.05 (0.94, 1.18)	Cognitive
Confusion**	111 (1.7)	613 (1)	0.93 (0.71, 1.21)	Cognitive
Emotional lability**	42 (0.7)	337 (0.5)	0.83 (0.55, 1.24)	Cognitive
Family history of MND**	19 (0.3)	10 (< 0.1)	18.04 (7.64, 42.60)	Supporting factor
Appetitie loss*	109 (1.7)	460 (0.7)	1.26 (0.94, 1.68)	Supporting factor
Weightloss	471 (7.3)	1685 (2.7)	2.20 (1.92, 2.52)	Literature
Ataxia	129 (2)	181 (0.3)	4.82 (3.58, 6.48)	Pilot study
Hoarseness of voice	434 (6.7)	1199 (1.9)	3.26 (2.82, 3.77)	Pilot study
Dysphasia	84 (1.3)	131 (0.2)	2.85 (1.92, 4.23)	Pilot study
Difficulty climbing stairs	24 (0.4)	107 (0.2)	1.46 (0.77, 2.75)	Pilot study
Wheeze	1064 (16.5)	7529 (12.1)	1.15 (1.01, 1.32)	Pilot study
Dry mouth	177 (2.7)	1082 (1.7)	0.97 (0.79, 1.20)	Pilot study
Constipation	958 (14.9)	5253 (8.5)	1.45 (1.31, 1.60)	Not supporting factor
Urinary incontinence	434 (6.7)	2542 (4.1)	1.28 (1.12, 1.47)	Not supporting factor
Nocturia	222 (3.4)	1581 (2.5)	1.08 (0.90, 1.30)	Not supporting factor
Urinary retention	148 (2.3)	942 (1.5)	1.06 (0.85, 1.33)	Not supporting factor
Urinary frequency increase	388 (6)	2982 (4.8)	0.84 (0.73, 0.97)	Not supporting factor

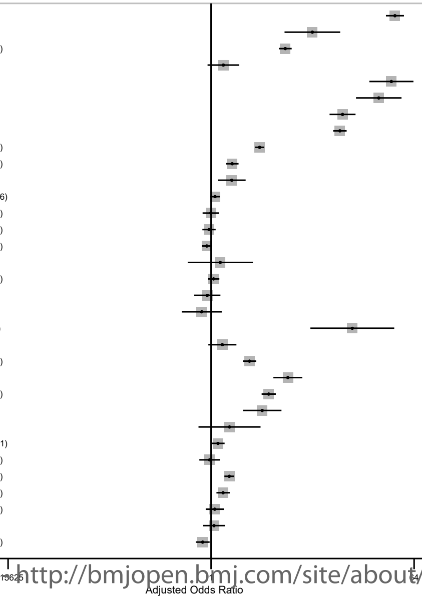

Model included signs and symptoms that were last recorded between 3 months to 5 years prior to MND diagnosis or index date

QResearch database version 44, 2020, University of Oxford, Mei et al

For peer review only - http://bmjopen.bmj.com/site/about/guidelines.xhtml

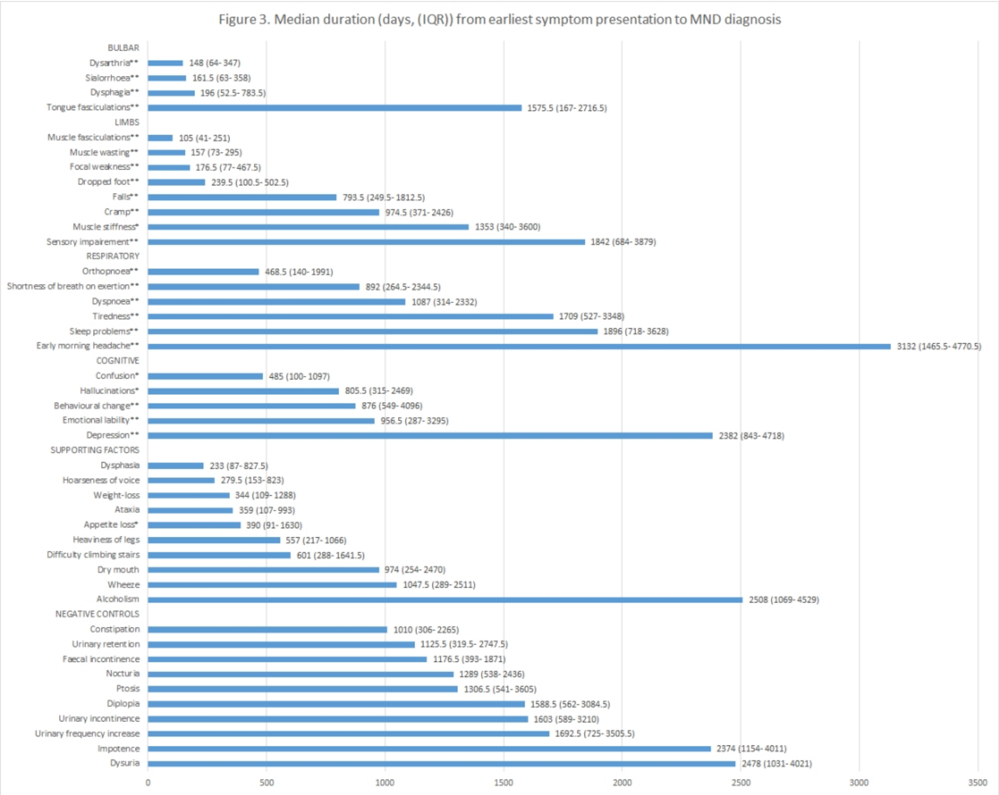

Median duration (days, (IQR)) from earliest symptom presentation to MND diagnosis

562x447mm (57 x 57 DPI)

STROBE Statement—Checklist of items that should be included in reports of ***case-control studies***

	Item No	Recommendation	Page No
**Title and abstract**	1	(*a*) Indicate the study's design with a commonly used term in the title or the abstract	1
		(*b*) Provide in the abstract an informative and balanced summary of what was done and what was found	2
**Introduction**			
Background/rationale	2	Explain the scientific background and rationale for the investigation being reported	3
Objectives	3	State specific objectives, including any prespecified hypotheses	3
**Methods**			
Study design	4	Present key elements of study design early in the paper	3
Setting	5	Describe the setting, locations, and relevant dates, including periods of recruitment, exposure, follow-up, and data collection	3
Participants	6	(*a*) Give the eligibility criteria, and the sources and methods of case ascertainment and control selection. Give the rationale for the choice of cases and controls	3
		(*b*) For matched studies, give matching criteria and the number of controls per case	4
Variables	7	Clearly define all outcomes, exposures, predictors, potential confounders, and effect modifiers. Give diagnostic criteria, if applicable	4
Data sources/ measurement	8*	For each variable of interest, give sources of data and details of methods of assessment (measurement). Describe comparability of assessment methods if there is more than one group	4 for both cases and controls
Bias	9	Describe any efforts to address potential sources of bias	5
Study size	10	Explain how the study size was arrived at	N/A
Quantitative variables	11	Explain how quantitative variables were handled in the analyses. If applicable, describe which groupings were chosen and why	4
Statistical methods	12	(*a*) Describe all statistical methods, including those used to control for confounding	5
		(*b*) Describe any methods used to examine subgroups and interactions	N/A
		(*c*) Explain how missing data were addressed	5
		(*d*) If applicable, explain how matching of cases and controls was addressed	
		(*e*) Describe any sensitivity analyses	
**Results**			
Participants	13*	(a) Report numbers of individuals at each stage of study—eg numbers potentially eligible, examined for eligibility, confirmed eligible, included in the study, completing follow-up, and analysed	5 for both cases and controls
		(b) Give reasons for non-participation at each stage	
		(c) Consider use of a flow diagram	
Descriptive data	14*	(a) Give characteristics of study participants (eg demographic, clinical, social) and information on exposures and potential confounders	5 for both cases and controls
		(b) Indicate number of participants with missing data for each variable of interest	5/Table1 for both

For peer review only - http://bmjopen.bmj.com/site/about/guidelines.xhtml

For peer review only

			cases and controls
Outcome data	15*	Report numbers in each exposure category, or summary measures of exposure	6/Figure1 for both cases and controls
Main results	16	(*a*) Give unadjusted estimates and, if applicable, confounder-adjusted estimates and their precision (eg, 95% confidence interval). Make clear which confounders were adjusted for and why they were included	6/Figure1
		(*b*) Report category boundaries when continuous variables were categorized	
		(*c*) If relevant, consider translating estimates of relative risk into absolute risk for a meaningful time period	
Other analyses	17	Report other analyses done—eg analyses of subgroups and interactions, and sensitivity analyses	
**Discussion**			
Key results	18	Summarise key results with reference to study objectives	6
Limitations	19	Discuss limitations of the study, taking into account sources of potential bias or imprecision. Discuss both direction and magnitude of any potential bias	7
Interpretation	20	Give a cautious overall interpretation of results considering objectives, limitations, multiplicity of analyses, results from similar studies, and other relevant evidence	
Generalisability	21	Discuss the generalisability (external validity) of the study results	
**Other information**			
Funding	22	Give the source of funding and the role of the funders for the present study and, if applicable, for the original study on which the present article is based	8

*Give information separately for cases and controls.

Note: An Explanation and Elaboration article discusses each checklist item and gives methodological background and published examples of transparent reporting. The STROBE checklist is best used in conjunction with this article (freely available on the Web sites of PLoS Medicine at http://www.plosmedicine.org/, Annals of Internal Medicine at http://www.annals.org/, and Epidemiology at http://www.epidem.com/). Information on the STROBE Initiative is available at http://www.strobe-statement.org.

For peer review only - http://bmjopen.bmj.com/site/about/guidelines.xhtml
For peer review only

BMJ Open

Identifying Key Signs of Motor Neurone Disease in Primary Care: A Nested Case-Control Study Using the QResearch Database

Journal:	*BMJ Open*
Manuscript ID	bmjopen-2021-058383.R1
Article Type:	Original research
Date Submitted by the Author:	12-Apr-2022
Complete List of Authors:	Mei, Xue; Oxford University, Nuffield Department of Primary Care Health Sciences<br>Burchardt, Judith; Oxford University, Primary Care Health Sciences<br>Ranger, Tom; Oxford University, Primary Care Health Sciences<br>McDermott, Christopher; The University of Sheffield<br>Radunovic, Aleksandar; Barts Health NHS Trust<br>Coupland, Carol; University of Oxford, Nuffield Department of Primary Care Health Sciences; University of Nottingham<br>Hippisley-Cox, Julia; University of Oxford, Nuffield Department of Primary Care Health Sciences
**Primary Subject Heading**:	Epidemiology
Secondary Subject Heading:	Neurology, Public health, General practice / Family practice
Keywords:	Motor neurone disease < NEUROLOGY, EPIDEMIOLOGY, PRIMARY CARE

SCHOLARONE™
Manuscripts

I, the Submitting Author has the right to grant and does grant on behalf of all authors of the Work (as defined in the below author licence), an exclusive licence and/or a non-exclusive licence for contributions from authors who are: i) UK Crown employees; ii) where BMJ has agreed a CC-BY licence shall apply, and/or iii) in accordance with the terms applicable for US Federal Government officers or employees acting as part of their official duties; on a worldwide, perpetual, irrevocable, royalty-free basis to BMJ Publishing Group Ltd ("BMJ") its licensees and where the relevant Journal is co-owned by BMJ to the co-owners of the Journal, to publish the Work in this journal and any other BMJ products and to exploit all rights, as set out in our licence.

The Submitting Author accepts and understands that any supply made under these terms is made by BMJ to the Submitting Author unless you are acting as an employee on behalf of your employer or a postgraduate student of an affiliated institution which is paying any applicable article publishing charge ("APC") for Open Access articles. Where the Submitting Author wishes to make the Work available on an Open Access basis (and intends to pay the relevant APC), the terms of reuse of such Open Access shall be governed by a Creative Commons licence – details of these licences and which Creative Commons licence will apply to this Work are set out in our licence referred to above.

Other than as permitted in any relevant BMJ Author's Self Archiving Policies, I confirm this Work has not been accepted for publication elsewhere, is not being considered for publication elsewhere and does not duplicate material already published. I confirm all authors consent to publication of this Work and authorise the granting of this licence.

For peer review only

TITLE

Identifying Key Signs of Motor Neurone Disease in Primary Care: A Nested Case-Control Study Using the QResearch Database

Xue W Mei[1], Judith Burchardt[1], Tom A Ranger[1], Christopher J McDermott[2], Aleksandar Radunovic[3], Carol Coupland[1,4], Julia Hippisley-Cox[1]

[1]Nuffield Department of Primary Care Health Sciences, Woodstock Road, University of Oxford

[2]Sheffield Institute for Translational Neuroscience, University of Sheffield, UK

[3]Barts MND Centre, Barts Health NHS Trust, UK

[4] School of Medicine, University of Nottingham, UK

Correspondence to: Professor Julia Hippisley-Cox, Nuffield Department of Primary Care Health Sciences, Radcliffe Observatory Quarter, Woodstock Road, Oxford julia.hippisley-cox@phc.ox.ac.uk @juliahcox

Word count: 3,490 excluding references

For peer review only

ABSTRACT

Objective To confirm the symptoms and signs for MND in the Red Flag tool; to quantify the extent to which the key symptoms and signs are associated with MND; and to identify additional factors which may be helpful within the primary care setting in recognition of possible MND and triggering timely referral to neurology specialists.

Design A nested case-control study.

Setting 1292 UK general practices contributing to the QResearch primary care database, linked to hospital and mortality data.

Participants Baseline cohort included 16.8 million individuals aged 18 years and over without a diagnosis of MND at study entry and with more than 3 years of digitalised information available. The nested case-control dataset comprised of 6,437 cases of MND diagnosed between January 1998 and December 2019, matched by year of birth, gender, general practice, and calendar year to 62,003 controls.

Main outcome measures Clinically recognised symptoms and signs of MND prior to diagnosis and symptoms and factors which are relevant in primary care setting.

Results This study identified 17 signs and symptoms that were independently associated with MND diagnosis in a multivariable analysis. Of these, 7 were new to the Red Flag tool: ataxia, dysphasia, weight-loss, wheeze, hoarseness of voice, urinary incontinence, and constipation. Amongst those from the Red Flag tool, dysarthria had the strongest association with subsequent MND (adjusted odds ratio (AOR): 43.5(95% CI 36.2 to 52.3)) followed by muscle fasciculations (AOR: 40.2 (95% CI 25.6 to 63.1)) and muscle wasting (AOR: 30.6 (95% CI 19.2 to 48.7)). Additionally, the associations between MND diagnosis and family history, dropped foot, focal weakness, and sialorrhoea remained robust after controlling for confounders. Patients that reported symptoms indicative of damage to the lower brainstem and its connections were diagnosed sooner than those who presented with respiratory or cognitive signs.

Conclusion This is the first study that has identified, confirmed, and quantified the association of key symptoms and signs with MND diagnosis. In addition to known factors, the study has identified the following new factors to be independently associated with MND prior to diagnosis: ataxia, dysphasia, wheeze, and hoarseness of voice. These findings may be used to improve risk stratification and earlier detection of MND in primary care.

(363 words)

For peer review only - http://bmjopen.bmj.com/site/about/guidelines.xhtml

STRENGTHS AND LIMITATIONS

- This is the largest nested-case control study derived from a representative population-based cohort looking at signs and symptoms related to MND
- We were able to collate an extensive list of signs and symptoms and to examine the association of each to MND diagnosis
- We were not able to determine the time point, number, and types of specialist referrals
- We do not know about completeness and accuracy of the recording, since not all patients with symptoms will seek medical attention and not all symptoms will be recorded at the GP consultations
- Some observed associations may be due to unmeasured confounding or variations in coding practices at the GPs

INTRODUCTION

Motor Neurone Disease (MND) causes progressive neuromuscular weakness that may first present as isolated and unexplained symptoms. MND causes the deaths of 1 in 350 men and 1 in 470 women in the UK[1], although there is emerging evidence that the incidence might be higher than previously thought[2]. In Europe, the annual crude incidence rate was estimated to be 2.7 per 100,000 person-years[3]. MND is difficult to recognise in primary care since it is both a relatively uncommon disease and the clinical presentations of early symptoms are sporadic and non-specific. As a result, patients may delay consulting their general practitioners (GPs) and the GPs may not attribute the symptoms to MND. Previous work has demonstrated that both patient factors (e.g. delayed presentation to GP) and health care practice factors (e.g. referrals to non-neurology specialists) contribute to delays in MND diagnosis hence patients' access to disease management[4]. The length of diagnostic delay have also been found to be mostly between 10-16 months from symptom onset to diagnosis[4].

In 2014, the Motor Neurone Disease Association and the Royal College of General Practitioners (RCGP) collaborated to produce a Red Flag tool for MND[5], which is designed to improve timely referrals to neurology specialists from primary care consultants. This tool was developed based on interviews with MND specialists and details a number of symptoms and signs which could be indicative of MND but has not been verified by analysis of clinical data. Therefore, the aims of our study are to confirm the symptoms and signs in the Red Flag tool; to quantify the extent to which the key symptoms and signs are associated with MND; as well as to identify additional factors which may be helpful within the primary care setting in recognition of possible MND and triggering timely referral to neurology specialists.

METHOD

Study design

We conducted a nested case-control study in a large UK primary care cohort using the QResearch database (version 44). QResearch is an anonymised, large open cohort database which prospectively collects routine health data from general practices in the UK that use the Egton Medical Information Systems (EMIS) computer system (used by 55% of GPs in the UK). The data include individual level data for demographics, lifestyle factors, medical diagnoses, prescriptions, specialist referrals, medical examinations, and examination results and are linked to Hospital Episode Statistics (HES) records and Office of National Statistics (ONS) death registry records.

For peer review only - http://bmjopen.bmj.com/site/about/guidelines.xhtml

Ascertainment of cases and controls

The base cohort included individuals aged 18 years and over registered during the study period (1 January 1998 to 31 December 2019) without a diagnosis of MND at study entry. The study entry date was defined as the latest of any of the following: 18th birthday; date of registration with the practice plus one year; date on which the practice computer system was installed plus one year; or the beginning of the study period (1 January 1998). The cohort was followed up until the earliest of any of the following: the date of MND diagnosis; date of 100th birthday; date of death; date of leaving the practice; or the study end date (31 December 2019).

Cases were defined as patients in the study cohort with a new diagnosis of MND on any of the GP record, Hospital Episode Statistics (HES) record or Office of National Statistics (ONS) death registry during follow-up. The following Read codes were used to identify the cases from the GP records: F152 (motor neurone disease), F1520 (amyotrophic lateral sclerosis (ALS)), F1521 (progressive muscular atrophy), F1522 (progressive bulbar palsy), F1523 (pseudobulbar palsy), F1524 (primary lateral sclerosis), and F152z (MND not otherwise specified). We also used ICD10 code G122 (motor neurone disease) to identify further records from HES and ONS death registry.

Controls were matched to cases by sex, practice, year of birth, and calendar year using the incidence density sampling method[6]. Each control was allocated an index date which was the date of first MND diagnosis of their matched cases. Each case was matched with up to 10 controls who were alive, registered, and without a diagnosis of MND at the index date. Cases and controls were excluded if they had less than three years of computerised data available at the index date, to ensure that the data are complete for a minimum of three years prior to index date.

Symptoms

Red flag symptoms included clinically recognised symptoms and signs of MND[5 7]. The list of existing factors are as following: bulbar features - dysarthria, dysphagia, sialorrhoea or excessive salivation, and tongue fasciculations; limb or muscle features - focal weakness, falls, foot drop, muscle wasting, muscle fasciculations or twitching, cramps, sensory impairment, and muscle stiffness; respiratory features - orthopnoea, dyspnoea, shortness of breath on exertion, sleep problems, tiredness or fatigue, and early morning headache; cognitive features – behavioural change, emotional lability, depression, hallucinations, and confusion; as well as appetite loss and family history of MND as supporting factors. Additionally, we included potential symptoms such as weight-loss, ataxia, dry mouth, heaviness of legs, and difficulty climbing stairs which were identified through literature searches[8-10] and a search of Read/SNOMED-CT codes for symptoms recorded prior to diagnosis in a pilot study involving a sample of patients diagnosed with MND in JB's general practice. These were included because although not directly established signs/symptoms of MND, these may provide valuable information in identifying additional patients who may benefit from neurologist referral in the primary care setting. We also included the following list of conditions to serve as negative controls since these are not usually considered to be associated with MND: diplopia, ptosis, bladder and bowel problems such as incontinence and constipation, as well as sexual dysfunction. All symptoms and signs were identified through searches of the GP records via corresponding Read/SNOMED-CT codes. For the primary analyses, only signs and symptoms that were last recorded between 3 months to 5 years prior to the index date were included, as symptoms occurring more than 5 years ago were considered unlikely to be relevant to MND and those recorded less than 3 months prior are likely to be reflective of the average waiting period between specialist referral and formal diagnosis. We also excluded very rare signs and symptoms (defined as < 5 ever recorded counts in either cases or

For peer review only - http://bmjopen.bmj.com/site/about/guidelines.xhtml

controls). Table 2 has a summary of the factors included in the analysis and the source of the information.

Statistical analysis

We estimated the strength of association of the red flag signs and symptoms with MND diagnosis, expressed as odds ratios (ORs), using conditional logistic regression. We performed both univariate and multivariable analysis, adjusted for the following confounders: the most recently recorded body mass index (BMI) prior to index date, latest records of smoking status and alcohol consumption, Townsend deprivation score in quintiles, and self-assigned ethnic groups. We assumed missing data on BMI, smoking, alcohol consumption, Townsend scores, and ethnic groups were missing at random and imputed them using multiple imputation by chained equations[11-13]. We created 20 imputed datasets and the imputation model included demographic variables, comorbidities or disease history (I.e. ever diagnosis of one or more of the following: cardiovascular diseases, stroke, diabetes (type 1 and 2), dementia, Parkinson's disease, myasthenia gravis, and multiple sclerosis), all symptoms and signs that were included in the final regression model, as well as the case-control indicator. The ORs from each imputed dataset were combined to form the final adjusted ORs using Rubin's rule[14]. Only signs and symptoms that reached statistical significance (two-tailed $P < 0.05$) in the univariate analysis were included in the multivariable analysis. All statistical analyses were performed using Stata16 (Stata Corp, College Station, TX).

Power calculations

A feasibility study identified over 5,000 patients with MND in the last 20 years in the QResearch. With 5000 cases and 10 matched controls per case we will be able to detect an odds ratio of 1.26 or more for a symptom recorded in 5% of controls, with 90% power and 1% significance. For a symptom recorded in 1% of controls we will be able to detect an odds ratio of 1.59 or more[15].

Patient and public involvement

This study was initiated by the Motor Neurone Disease Association which is a charity representing patients with MND. Apart from this, patients and public were not involved in design and development of the study or interpretation of the study outcome but will be consulted regarding the dissemination of the results.

RESULTS

Descriptive characteristics of the study cohort

The base cohort comprised of 16,799,992 participants aged between 18 and 100 years. During a total of 112,003,453 person-years of follow-up, we found 6,437 incident cases of MND that were eligible for inclusion, using records from general practice, hospital episode statistics, and death registry databases. We were able to identify a total of 62,003 birth year, gender, practice, and calendar year matched controls. 56.8% of the MND cases were men and mean (SD) age at diagnosis was 69.6 (12.2) years.

Table 1 is a summary of the demographic characteristics of the cases and controls. The ethnicity recording was more complete in controls (71.3%) than in cases (55.4%), however, completeness of Townsend deprivation score, alcohol, smoking, and BMI were similar between the two groups. There was no significant difference between cases and controls in terms of alcohol consumption or smoking status. However, compared with the controls, the cases had lower BMI ($P < 0.001$) and were more likely to be living in less deprived areas based on Townsend score quintiles ($P = 0.013$).

For peer review only - http://bmjopen.bmj.com/site/about/guidelines.xhtml

Table 1. Demographic characteristics of case patients with motor neurone disease and age, sex practice, calendar time matched controls. Figures are numbers (%) unless otherwise indicated.

Characteristic	Study Participants, No. (column %)	
	controls (n = 62,003)	cases (n= 6,437)
Women	27058(43.6)	2781(43.2)
Men	34945(56.4)	3656(56.8)
Age at diagnosis/index date (mean(SD)), y	69.1(11.9)	69.6(12.2)
Age at study entry		
< 50	10700(17.3)	1078(16.7)
50 - 54	6556(10.6)	651(10.1)
55 - 59	8016(12.9)	793(12.3)
60 - 64	9678(15.6)	976(15.2)
65 - 69	9734(15.7)	982(15.3)
70 - 74	8085(13)	852(13.2)
75 - 79	5771(9.3)	627(9.7)
80 +	3463(5.6)	478(7.4)
Ethnicity recorded	44211 (71.3)	3563 (55.4)
**Ethnicity**		
White	41106(66.3)	3281(51)
Indian	700(1.1)	69(1.1)
Pakistani	349(0.6)	32(0.5)
Bangladeshi	174(0.3)	19(0.3)
Other Asian	322(0.5)	30(0.5)
Caribbean	537(0.9)	48(0.7)
Black African	373(0.6)	28(0.4)
Chinese	132(0.2)	4(0.1)
Other	518(0.8)	52(0.8)
Not recorded	17792(28.7)	2874(44.6)
**Townsend deprivation score, quintiles**		
1 (least deprived)	20410(32.9)	2205(34.3)
2	16131(26)	1613(25.1)
3	11533(18.6)	1174(18.2)

For peer review only

4	8263(13.3)	795(12.4)
5 (most deprived)	5587(9)	640(9.9)
Townsend deprivation score not recorded	79(0.1)	10(0.2)
BMI not recorded before index date	11352(18.3)	1124 (17.5)
BMI (mean(SD))	26.6(4.6)	26.2(4.5)
**Smoking status**		
Non-smoker	29655(47.8)	3142(48.8)
Ex-smoker	17756(28.6)	1882(29.2)
Light smoker (1-9 cigarettes/day)	5931(9.6)	596(9.3)
Moderate smoker (10-19 cigarettes/day)	1125(1.8)	109(1.7)
Heavy smoker (≥20 cigarettes/day)	816(1.3)	76(1.2)
Smoking not recorded before index date	6720(10.8)	632(9.8)
**Alcohol consumption**		
Non-drinker	31677(51.1)	3454(53.7)
Trivial (<1U/day)	9215(14.9)	906(14.1)
Light (1-2u/day)	4460(7.2)	465(7.2)
Moderate (3-6u/day)	4582(7.4)	453(7)
Heavy (7-9u/day)	343(0.6)	30(0.5)
Very Heavy (>9u/day)	129(0.2)	15(0.2)
Alcohol not recorded before index date	11597(18.7)	1114(17.3)

Association of red flag signs and symptoms to MND diagnosis

The types of symptoms and signs as well as their adjusted estimates are presented in Figure 1 (univariate) and Figure 2 (multivariate). Table 2 also contains the list of factors examined, source from which they were obtained, and whether they were statistically significant in the analysis. 33 signs and symptoms reached our pre-specified level of statistical significance (two-tailed $P < 0.05$) in the unadjusted univariate analysis, and these remained significant after adjustment for BMI, alcohol consumption, smoking, deprivation, and self-reported ethnicity (Figure 1). These were collectively included in a multivariable model for estimation of their independent associations with MND diagnosis. Dysarthria was the strongest independent predictor of MND diagnosis (OR 43.2, 95%CI 36.0-52.0) followed by muscle fasciculations (OR (95%CI) 40.2 (25.6-63.1)), muscle wasting (OR (95%CI) 31.0 (19.5-49.4)), and dropped foot (OR (95%CI) 14.8 (11.3-19.3)). Some respiratory symptoms such as shortness of breath and dyspnoea, as well as cognitive symptoms such as depression and confusion, though statistically significant in univariate analysis, were no longer associated with MND after controlling for other symptoms and signs. We also found the following symptoms, which were not included in the RF tool but were found in GP records, to be independently associated with MND diagnosis: hoarseness of voice (OR (95%CI) 3.26 (2.82-3.77)), dysphasia (OR

For peer review only - http://bmjopen.bmj.com/site/about/guidelines.xhtml

(95%CI) 2.85 (1.92-4.23)), ataxia (OR (95%CI) 4.82 (3.58-6.48)), wheeze (OR (95%CI) 1.15 (1.01-1.32)), and weight-loss (OR (95%CI) 2.20 (1.92-2.52)).

Table 2 Factors included for analysis and the source from which they were identified[1]

	**Symptoms and signs**	**Source/Reason for inclusion**	**Confirmed on analysis[2]**
**Bulbar features**			
	Dysarthria	MNDA	UV, MV
	Dysphagia	MNDA	UV, MV
	Sialorrhoea/excessive salivation	MNDA	UV, MV
	Tongue fasciculations	MNDA	UV
**Limb Features/muscle weakness**			
	Focal weakness	MNDA	UV, MV
	Falls	MNDA	UV, MV
	Foot drop	MNDA	UV, MV
	Muscle wasting	MNDA	UV, MV
	Muscle fasciculations or twitching	MNDA	UV, MV
	Cramps	MNDA	UV, MV
	Sensory impairment	MNDA	No
	Muscle stiffness	NICE	UV, MV
**Respiratory features**			
	Orthopnoea	MNDA	No
	Dyspnoea	MNDA	UV
	Shortness of breath on exertion	MNDA	UV
	Sleep problems	MNDA	UV
	Tiredness/fatigue	MNDA	UV
	Early morning headache	MNDA	No
**Cognitive features**			
	Behavioural change	MNDA	No
	Emotional lability	MNDA	UV
	Depression	MNDA	UV
	Hallucinations	NICE	UV
	Confusion	NICE	UV
**Supporting factors**			
	Family history of MND	MNDA	UV, MV
	Appetite loss	NICE	UV

For peer review only - http://bmjopen.bmj.com/site/about/guidelines.xhtml

**Additional factors from literature review/pilot study**			
	Alcoholism	Literature	No
	Weight-loss	Literature	UV, MV
	Ataxia	Pilot study	UV, MV
	Wheeze	Pilot study	UV, MV
	Difficulty climbing stairs	Pilot study	UV
	Dry mouth	Pilot study	UV
	Dysphasia	Pilot study	UV, MV
	Hoarseness of voice	Pilot study	UV, MV
**Factors not considered supportive of diagnosis by MNDA**			
	Urinary frequency increase	Negative control	UV
	Urinary incontinence	Negative control	UV, MV
	Urinary retention	Negative control	UV
	Nocturia	Negative control	UV
	Dysuria	Negative control	No
	Constipation	Negative control	UV, MV
	Faecal incontinence	Negative control	No
	Impotence	Negative control	No
	Diplopia	Negative control	No
	Ptosis	Negative control	No

[1] The study was matched on age and gender therefore they were not included in the analysis model

[2] Indicates the analysis model used to find the associations. All models were additionally adjusted for Body Mass Index, Townsend scores in quintiles, smoking status, alcohol consumption behaviour, and self-reported ethnicity; UV Univariate, MV Multivariate

(Figure 1)

We also observed independent associations with MND for two of the symptoms not generally considered to be associated with MND: constipation (OR (95%CI) 1.45 (1.31-1.60)) and urinary incontinence (OR (95%CI) = 1.28 (1.12-1.47)).

Overall, symptoms from bulbar dysfunction (i.e. dysarthria, dysphagia, sialorrhoea, and tongue fasciculations) and those appearing in the limbs are most strongly suggestive of subsequent MND diagnosis. Symptoms and signs occurring in the respiratory and cognitive systems were not statistically significant when evaluated in combination with others.

For peer review only - http://bmjopen.bmj.com/site/about/guidelines.xhtml

(Figure 2)

Time from earliest symptom presentation to MND diagnosis

Duration from the earliest symptom presentation to MND diagnosis varied considerably by feature and location of the signs and symptoms. Patients presenting with symptoms and signs associated with damage of bulbar region and limb functions received their diagnoses sooner than those presenting with respiratory or cognitive symptoms and signs (e.g. median days from symptom presentation to diagnosis: dysarthria 145.5 days (IQR 68.5 – 296 days) vs. orthopnoea 299 days (IQR 42 – 496 days)). Amongst those clinical features, unusual and sporadic signs such as dysarthria and muscle fasciculations appeared to be the key indicators to speedy neurologist referral. Time from earliest clinical symptom presentation to MND diagnosis (median days, IQR) is presented in Figure 3.

(Figure 3)

DISCUSSION

Principal findings

In this very large population based nested case-control study covering a population of over 16 million adults, we were able to demonstrate that the Motor Neurone Disease Association recommended Red Flag signs and symptoms[5], particularly those affecting bulbar and limb regions, are independently predictive of subsequent MND diagnosis. Amongst our study of 6,437 MND patients and 62,003 matched controls, the symptom with the largest association with MND was dysarthria followed by muscle fasciculations and muscle wasting. Additionally from the GP records, we found the following symptoms to be independently associated with MND: hoarseness of voice, dysphasia, ataxia, wheeze, and weight-loss. We also found a higher proportion of MND cases with documented constipation and urinary incontinence than the controls.

Consistent with previous studies[16 17], we found patients showing bulbar onset symptoms such as dysarthria and dysphagia were diagnosed on average 4-5 months sooner than those with limb onset symptoms. Interestingly in our cohort, patients presenting with muscle fasciculations were diagnosed most quickly, which is likely as a result of timely and adequate specialist referral by their managing GPs. This is consistent with a qualitative study conducted by Baxter and McDermott[18] which explored the decision-making and referral process of GPs through semi-structured interviews. The study found that among the 42 GPs who consented to participate, fasciculation was the most commonly described trigger for suspected MND and neurologist referral[18]. Our finding demonstrated that muscle fasciculation is indeed likely one of the top triggers for specialist referral practised by GPs across England. We found the patients who exhibited speech related symptoms such as dysarthria received a diagnosis within an average of 6 months of symptom presentation. This suggests that the awareness of speech impairment in neurological disorders is relatively high amongst GPs in England, in contrast to previous studies[18].

Signs and symptoms of respiratory and cognitive systems, while moderately associated with MND in univariate analysis, were no longer significant after accounting for symptoms related to bulbar region impairment and limb functions. The period between initial presentation of respiratory and cognitive symptoms and MND diagnosis is also significantly longer. This finding seems to indicate that presentations of respiratory or cognitive featured symptoms alone were insufficient to trigger a specialist referral. One reason for this may be that the symptom onset is relatively mild and sporadic,

For peer review only - http://bmjopen.bmj.com/site/about/guidelines.xhtml

and may not alarm the managing GPs since many other patients also have comorbidities with similar symptom profile. However, GPs could cross reference these symptoms against others (i.e. dysarthria or muscle wasting) to enable neurologist referral.

The positive associations between constipation and urinary incontinence with MND found in our study were unexpected since these have not been described in MND before[9 19]. We suspect the observed effect is primarily due to reverse causality: patients with undiagnosed but advanced MND are likely to have trouble with balance and movement, thereby hindering their ability to perform tasks such as going to the toilet.

Strengths and limitations

This study is to date, the largest nested-case control study derived from a representative population-based cohort looking at signs and symptoms related to MND. We were able to collate an extensive list of signs and symptoms from the GP records, and to examine the association of each sign and symptom to subsequent MND diagnosis. We were also able to demonstrate that duration of the earliest symptom presentation to MND diagnosis could serve as an indicator to key signs and symptoms that triggered neurologist referral. We could not determine the time point, number, and types of specialist referrals because our study was designed to compare the pattern of symptoms prior to diagnosis in cases (and the equivalent index date for controls). Another important limitation to our study is the completeness and accuracy of the recording of the signs and symptoms, since not all patients with symptoms will seek medical attention and not all symptoms will be recorded at the GP consultations. Furthermore, the positive associations may be attributed to unmeasured confounding or variation in coding practices at the GPs, due to the nature of routinely collected data. For example, we found dysphasia and wheeze to be independently and positively associated with MND. However these symptoms are not traditionally considered within the clinical features of MND although dysphasia has been described to present in ALS in a systematic review[20]. It might also be that the patients presented with both dysarthria (disorder of speech) and dysphasia (disorder of language) but only dysphasia was coded because it was the dominant symptom when GP consultation was sought. Similarly, shortness of breath on exertion is a well-recognised sign of MND and might be coded as wheeze if symptom presentation of respiratory function tests are not undertaken.

CONCLUSION

In this very large population based nested case-control study of 6,437 MND cases and 62,003 matched controls, we were able to confirm the currently recognised signs and symptoms, particularly those of bulbar and limb regions to be independently associated with MND. Additionally, we found GP recorded hoarseness of voice, dysphasia, ataxia, and weight-loss to be associated with subsequent MND diagnosis. Due to the nature of the data as highlighted in the limitations, we could not verify whether all positive findings were true symptoms caused by MND or whether they were due to patient presentation patterns or GP coding behaviour. However, these findings have implications for GPs when considering referrals to neurologists and may be used to improve risk stratification and prediction of MND in primary care.

Future studies may focus on chronologically mapping the symptoms, characterising the patterns and clusters in symptoms presentation, and predicting disease progression based on types and duration of existing symptoms.

CONTRIBUTORS

For peer review only - http://bmjopen.bmj.com/site/about/guidelines.xhtml

XM undertook data manipulation, reviewed the literature, led the data analysis, undertook interpretation of the data, and wrote the first draft of the paper. JHC led the study, obtained funding, data approvals, designed the study, drafted the protocol, contributed to the data management and interpretation of the data. CJM contributed to the funding application, contributed to protocol development and interpretation of results. CC contributed to the funding application, the discussion on protocol development and provided critical feedback on drafts of the manuscript. JB reviewed the literature on presentation of MND and conducted a search of how it had presented in patients in her own general practice. TR contributed to interpretation of findings and compilation of the manuscript. XM, JHC, CC, CJM, JB, AR, and TR approved the protocol, contributed to the critical revision of the manuscript and approved the final version of the manuscript. The corresponding author attests that all listed authors meet authorship criteria and that no others meeting the criteria have been omitted.

For peer review only

FUNDING

This study was funded by a grant from Motor Neurone Disease Association. The funders of this study contributed to the design and conduct of the study and reviewed and approve the manuscript. The funders had no role in analysis or interpretation of data. XM, TR, CC, JHC had full access to all the study data and JHC had final responsibility for submission.

COMPETING INTEREST

All authors have completed the ICMJE uniform disclosure form at www.icmje.org/coi_disclosure.pdf. JHC reports grants from grants from the John Fell Oxford University Press Research Fund; Cancer Research UK (CR-UK) grant number C5255/A18085, through the Cancer Research UK Oxford Centre and the Oxford Wellcome Institutional Strategic Support Fund (204826/Z/16/Z), during the conduct of the study. JHC is an unpaid director of QResearch, a not-for-profit organisation which is a partnership between the University of Oxford and EMIS Health who supply the QResearch database used for this work. JHC is a founder and shareholder of ClinRisk ltd and was its medical director until 31st May 2019. JHC is member of the SAGE subgroups on ethnicity and data and is chair of the risk stratification subgroup of NERVTAG. The views expressed are those of the authors only. CJM is supported by the NIHR Sheffield Biomedical Centre.

ETHICAL APPROVAL

The QResearch® ethics approval is with East Midlands-Derby Research Ethics Committee [reference 18/EM/0400, project reference OX1].

DATA SHARING

To guarantee the confidentiality of personal and health information only the authors have had access to the data during the study in accordance with the relevant licence agreements. Access to the QResearch data is according to the information on the QResearch website (www.qresearch.org).

TRANSPARENCY

For peer review only - http://bmjopen.bmj.com/site/about/guidelines.xhtml

XM, TR, and JHC had full access to all data in the study and takes responsibility for the integrity of the data and the accuracy of the data analysis. JHC is the guarantor for the study and affirms that the manuscript is an honest, accurate, and transparent account of the study reported; that no important aspects of the study have been omitted; and that any discrepancies from the study as planned have been explained.

DISSEMINATION TO PARTICIPANTS AND RELATED PATIENT AND PUBLIC COMMUNITIES

PROVENANCE AND PEER REVIEW

Not commissioned; externally peer reviewed.

COPYRIGHT STATEMENT/LICENCE FOR PUBLICATION

"I, the Submitting Author has the right to grant and does grant on behalf of all authors of the Work (as defined in the author licence), an exclusive licence on a worldwide, perpetual, irrevocable, royalty-free basis to BMJ Publishing Group Ltd ("BMJ") its licensees. The Submitting Author accepts and understands that any supply made under these terms is made by BMJ to the Submitting Author unless I am acting as an employee on behalf of my employer which is paying any applicable article publishing charge ("APC") for Open Access articles. Where the Submitting Author wishes to make the Work available on an Open Access basis (and intends to pay the relevant APC), the terms of reuse of such Open Access shall be governed by a Creative Commons licence – details of these licences and which licence will apply to this Work are set out in our licence referred to above."

For peer review only

For peer review only - http://bmjopen.bmj.com/site/about/guidelines.xhtml

Figure 1. Univariate analysis for risk of MND diagnosis based on 6,437 cases and 62,003 age, sex, practice matched controls adjusted for variables shown, age, BMI, alcohol, smoking, ethnicity, deprivation

Figure 2. Multivariate analysis for risk of MND diagnosis based on 6,437 cases and 62,003 age, sex, practice matched controls adjusted for variables shown, age, BMI, alcohol, smoking, ethnicity, deprivation

Figure 3. Median duration (days, (IQR)) from earliest symptom presentation to MND diagnosis

For peer review only

REFERENCES

1. Alonso A, Logroscino G, Jick SS, et al. Incidence and lifetime risk of motor neuron disease in the United Kingdom: a population-based study. *Eur J Neurol* 2009;16(6):745-51.
2. Burchardt JM, Mei XW, Ranger T, et al. Analysis of incidence of motor neuron disease in England 1998–2019: use of three linked datasets. *Amyotrophic Lateral Sclerosis and Frontotemporal Degeneration* 2022:1-9. doi: 10.1080/21678421.2021.2016837
3. Logroscino G, Traynor BJ, Hardiman O, et al. Incidence of amyotrophic lateral sclerosis in Europe. *J Neurol Neurosurg Psychiatry* 2010;81(4):385-90. doi: 10.1136/jnnp.2009.183525 [published Online First: 2009/08/28]
4. Richards D, Morren JA, Pioro EP. Time to diagnosis and factors affecting diagnostic delay in amyotrophic lateral sclerosis. *Journal of the Neurological Sciences* 2020;417:117054. doi: https://doi.org/10.1016/j.jns.2020.117054
5. Association MND. Red Flag Diagnosis Tool [Available from: https://www.mndassociation.org/app/uploads/2013/10/red-flags-final-2.pdf accessed July 2020.
6. Vandenbroucke JP, Pearce N. Case–control studies: basic concepts. *International Journal of Epidemiology* 2012;41(5):1480-89. doi: 10.1093/ije/dys147
7. (NICE) NIfHaCE. Motor neurone disease: assessment and management 2016 [Available from: https://www.nice.org.uk/guidance/ng422019.
8. Kiernan MC, Vucic S, Cheah BC, et al. Amyotrophic lateral sclerosis. *The Lancet* 2011;377(9769):942-55. doi: 10.1016/S0140-6736(10)61156-7
9. McDermott CJ, Shaw PJ. Diagnosis and management of motor neurone disease. *BMJ* 2008;336(7645):658. doi: 10.1136/bmj.39493.511759.BE
10. Nageshwaran S, Davies LM, Rafi I, et al. Motor neurone disease. *BMJ : British Medical Journal* 2014;349
11. Group TAM. Academic Medicine: problems and solutions. *British Medical Journal* 1989;298:573-79.
12. Schafer J, Graham J. Missing data: our view of the state of the art. *Psychological Methods* 2002;7:147-77.
13. Steyerberg EW, van Veen M. Imputation is beneficial for handling missing data in predictive models. *J Epidemiol Community Health* 2007;60:979.
14. Rubin DB. Multiple Imputation for Non-response in Surveys. New York: John Wiley 1987.
15. Dupont WD. Power Calculations for Matched Case-Control Studies. *Biometrics* 1988;44(4):1157-68. doi: 10.2307/2531743
16. Galvin M, Ryan P, Maguire S, et al. The path to specialist multidisciplinary care in amyotrophic lateral sclerosis: A population- based study of consultations, interventions and costs. *PloS one* 2017;12(6):e0179796-e96. doi: 10.1371/journal.pone.0179796
17. Kraemer M, Buerger M, Berlit P. Diagnostic problems and delay of diagnosis in amyotrophic lateral sclerosis. *Clin Neurol Neurosurg* 2010;112(2):103-5. doi: 10.1016/j.clineuro.2009.10.014 [published Online First: 2009/11/26]
18. Baxter S, McDermott CJ. Decision-making and referral processes for patients with motor neurone disease: a qualitative study of GP experiences and evaluation of a new decision-support tool. *BMC Health Serv Res* 2017;17(1):339. doi: 10.1186/s12913-017-2286-0 [published Online First: 2017/05/10]
19. Foster LA, Salajegheh MK. Motor Neuron Disease: Pathophysiology, Diagnosis, and Management. *The American Journal of Medicine* 2019;132(1):32-37. doi: https://doi.org/10.1016/j.amjmed.2018.07.012

For peer review only - http://bmjopen.bmj.com/site/about/guidelines.xhtml

20. Pinto-Grau M, Hardiman O, Pender N. The Study of Language in the Amyotrophic Lateral Sclerosis - Frontotemporal Spectrum Disorder: a Systematic Review of Findings and New Perspectives. *Neuropsychology Review* 2018;28(2):251-68. doi: 10.1007/s11065-018-9375-7

For peer review only

For peer review only - http://bmjopen.bmj.com/site/about/guidelines.xhtml

Figure 1. Univariate analysis for risk of MND diagnosis based on 6,437 cases and 62,003 age, sex, practice matched controls

adjusted for variables shown, age, BMI, alcohol, smoking, ethnicity, deprivation

Symptoms	Cases	Controls	OR (95% CI)	Feature
Dysarthria**	362 (5.6)	124 (0.2)	36.51 (29.27, 45.54)	Bulbar
Sialorrhoea**	29 (0.5)	21 (< 0.1)	12.95 (7.29, 23.03)	Bulbar
Dysphagia**	295 (4.6)	544 (0.9)	6.50 (5.62, 7.52)	Bulbar
Tongue fasciculations**	35 (0.5)	181 (0.3)	1.83 (1.27, 2.65)	Bulbar
Muscle fasciculations**	65 (1)	15 (< 0.1)	50.90 (28.22, 91.80)	Limb
Muscle wasting**	53 (0.8)	21 (< 0.1)	25.69 (15.28, 43.19)	Limb
Dropped foot**	129 (2)	68 (0.1)	20.24 (14.97, 27.37)	Limb
Focal weakness**	364 (5.7)	351 (0.6)	12.19 (10.42, 14.26)	Limb
Falls**	846 (13.1)	3217 (5.2)	3.57 (3.26, 3.92)	Limb
Cramp**	318 (4.9)	1283 (2.1)	2.56 (2.25, 2.91)	Limb
Muscle stiffness*	39 (0.6)	170 (0.3)	2.41 (1.71, 3.41)	Limb
Sensory impairement**	120 (1.9)	1036 (1.7)	1.17 (0.97, 1.42)	Limb
Nocturia	146 (2.3)	841 (1.4)	1.67 (1.39, 2.00)	Respiratory
Shortness of breath**	443 (6.9)	3070 (5)	1.53 (1.37, 1.70)	Respiratory
Tiredness**	470 (7.3)	3251 (5.2)	1.48 (1.33, 1.64)	Respiratory
Dyspnoea**	550 (8.5)	3927 (6.3)	1.47 (1.33, 1.62)	Respiratory
Orthopnoea**	9 (0.1)	66 (0.1)	1.31 (0.65, 2.66)	Respiratory
Sleep problems**	443 (6.9)	3443 (5.6)	1.29 (1.16, 1.44)	Respiratory
Early morning headache**	338 (5.3)	3018 (4.9)	1.11 (0.99, 1.25)	Respiratory
Hallucinations*	13 (0.2)	43 (0.1)	2.55 (1.30, 5.00)	Cognitive
Emotional lability**	26 (0.4)	137 (0.2)	1.95 (1.28, 2.96)	Cognitive
Depression**	263 (4.1)	1521 (2.5)	1.81 (1.57, 2.07)	Cognitive
Confusion*	70 (1.1)	433 (0.7)	1.54 (1.19, 1.99)	Cognitive
Behavioural change**	14 (0.2)	79 (0.1)	1.53 (0.86, 2.74)	Cognitive
Family history of MND**	19 (0.3)	10 (< 0.1)	19.04 (8.80, 41.18)	Supporting factor
Appetite loss*	55 (0.9)	279 (0.4)	1.94 (1.44, 2.61)	Supporting factor
Weightloss	254 (3.9)	910 (1.5)	2.93 (2.53, 3.39)	Literature
Alcoholism	43 (0.7)	357 (0.6)	1.21 (0.88, 1.67)	Literature
Dysphasia	48 (0.7)	52 (0.1)	9.78 (6.49, 14.74)	Pilot study
Ataxia	71 (1.1)	95 (0.2)	7.83 (5.73, 10.71)	Pilot study
Hoarseness of voice	274 (4.3)	518 (0.8)	5.47 (4.69, 6.38)	Pilot study
Difficulty climbing stairs	17 (0.3)	81 (0.1)	2.24 (1.25, 4.00)	Pilot study
Dry mouth	100 (1.6)	575 (0.9)	1.68 (1.35, 2.09)	Pilot study
Wheeze	609 (9.5)	4459 (7.2)	1.46 (1.33, 1.61)	Pilot study
Constipation	628 (9.8)	3119 (5)	2.17 (1.97, 2.38)	Not supporting factor
Urinary incontinence	244 (3.8)	1237 (2)	2.01 (1.74, 2.33)	Not supporting factor
Urinary retention	84 (1.3)	508 (0.8)	1.56 (1.23, 1.98)	Not supporting factor
Ptosis	17 (0.3)	118 (0.2)	1.55 (0.95, 2.53)	Not supporting factor
Faecal incontinence	11 (0.2)	65 (0.1)	1.52 (0.79, 2.91)	Not supporting factor
Diplopia	30 (0.5)	201 (0.3)	1.46 (0.99, 2.14)	Not supporting factor
Urinary frequency increase	214 (3.3)	1515 (2.4)	1.38 (1.19, 1.61)	Not supporting factor
Impotence	251 (3.9)	2378 (3.8)	1.04 (0.91, 1.20)	Not supporting factor
Dysuria	103 (1.6)	984 (1.6)	0.98 (0.80, 1.21)	Not supporting factor

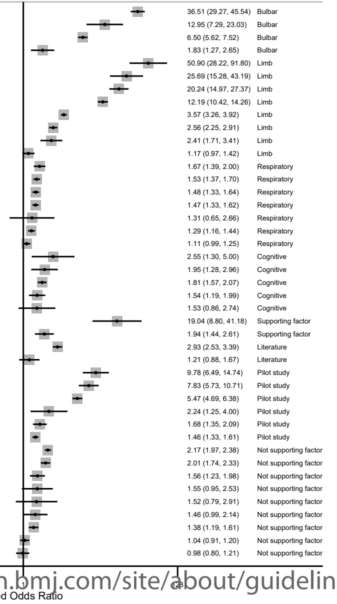

For peer review only - http://bmjopen.bmj.com/site/about/guidelines.xhtml

Model included signs and symptoms that were last recorded between 3 months to 5 years prior to MND diagnosis or index date

QResearch database version 44, 2020, University of Oxford, Mei et al

Figure 2. Multivariate analysis for risk of MND diagnosis based on 6,437 cases and 62,003 age, sex, practice matched controls

adjusted for variables shown, age, BMI, alcohol, smoking, ethnicity, deprivation

Symptoms	Cases	Controls	OR (95% CI)	Feature
Dysarthria**	790 (12.3)	214 (0.3)	43.22 (35.95, 51.97)	Bulbar
Sialorrhoea**	54 (0.8)	35 (0.1)	7.96 (4.50, 14.08)	Bulbar
Dysphagia**	652 (10.1)	1110 (1.8)	4.57 (4.01, 5.21)	Bulbar
Tongue fasciculations**	76 (1.2)	363 (0.6)	1.29 (0.93, 1.78)	Bulbar
Muscle fasciculations**	134 (2.1)	34 (0.1)	40.19 (25.59, 63.13)	Limb
Muscle wasting**	102 (1.6)	39 (0.1)	31.02 (19.46, 49.44)	Limb
Dropped foot**	208 (3.2)	126 (0.2)	14.78 (11.33, 19.28)	Limb
Focal weakness**	736 (11.4)	599 (1)	14.00 (12.20, 16.07)	Limb
Falls**	1304 (20.3)	5290 (8.5)	2.70 (2.46, 2.97)	Limb
Cramp**	470 (7.3)	2427 (3.9)	1.54 (1.35, 1.76)	Limb
Muscle stiffness*	84 (1.3)	446 (0.7)	1.53 (1.15, 2.03)	Limb
Tiredness**	903 (14)	6545 (10.6)	1.08 (0.98, 1.20)	Respiratory
Shortness of breath**	728 (11.3)	5031 (8.1)	1.00 (0.84, 1.18)	Respiratory
Dyspnoea**	819 (12.7)	5990 (9.7)	0.96 (0.84, 1.10)	Respiratory
Sleep problems**	789 (12.3)	6151 (9.9)	0.92 (0.83, 1.02)	Respiratory
Hallucinations*	22 (0.3)	74 (0.1)	1.21 (0.62, 2.35)	Cognitive
Depression**	545 (8.5)	4001 (6.5)	1.05 (0.94, 1.18)	Cognitive
Confusion**	111 (1.7)	613 (1)	0.93 (0.71, 1.21)	Cognitive
Emotional lability**	42 (0.7)	337 (0.5)	0.83 (0.55, 1.24)	Cognitive
Family history of MND**	19 (0.3)	10 (< 0.1)	18.04 (7.64, 42.60)	Supporting factor
Appetitie loss*	109 (1.7)	460 (0.7)	1.26 (0.94, 1.68)	Supporting factor
Weightloss	471 (7.3)	1685 (2.7)	2.20 (1.92, 2.52)	Literature
Ataxia	129 (2)	181 (0.3)	4.82 (3.58, 6.48)	Pilot study
Hoarseness of voice	434 (6.7)	1199 (1.9)	3.26 (2.82, 3.77)	Pilot study
Dysphasia	84 (1.3)	131 (0.2)	2.85 (1.92, 4.23)	Pilot study
Difficulty climbing stairs	24 (0.4)	107 (0.2)	1.46 (0.77, 2.75)	Pilot study
Wheeze	1064 (16.5)	7529 (12.1)	1.15 (1.01, 1.32)	Pilot study
Dry mouth	177 (2.7)	1082 (1.7)	0.97 (0.79, 1.20)	Pilot study
Constipation	958 (14.9)	5253 (8.5)	1.45 (1.31, 1.60)	Not supporting factor
Urinary incontinence	434 (6.7)	2542 (4.1)	1.28 (1.12, 1.47)	Not supporting factor
Nocturia	222 (3.4)	1581 (2.5)	1.08 (0.90, 1.30)	Not supporting factor
Urinary retention	148 (2.3)	942 (1.5)	1.06 (0.85, 1.33)	Not supporting factor
Urinary frequency increase	388 (6)	2982 (4.8)	0.84 (0.73, 0.97)	Not supporting factor

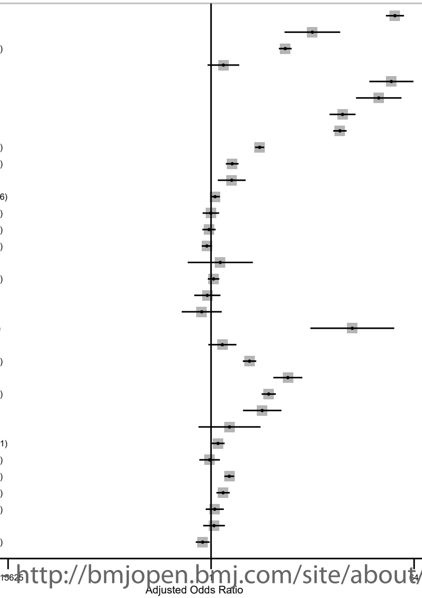

Model included signs and symptoms that were last recorded between 3 months to 5 years prior to MND diagnosis or index date

QResearch database version 44, 2020, University of Oxford, Mei et al

For peer review only - http://bmjopen.bmj.com/site/about/guidelines.xhtml

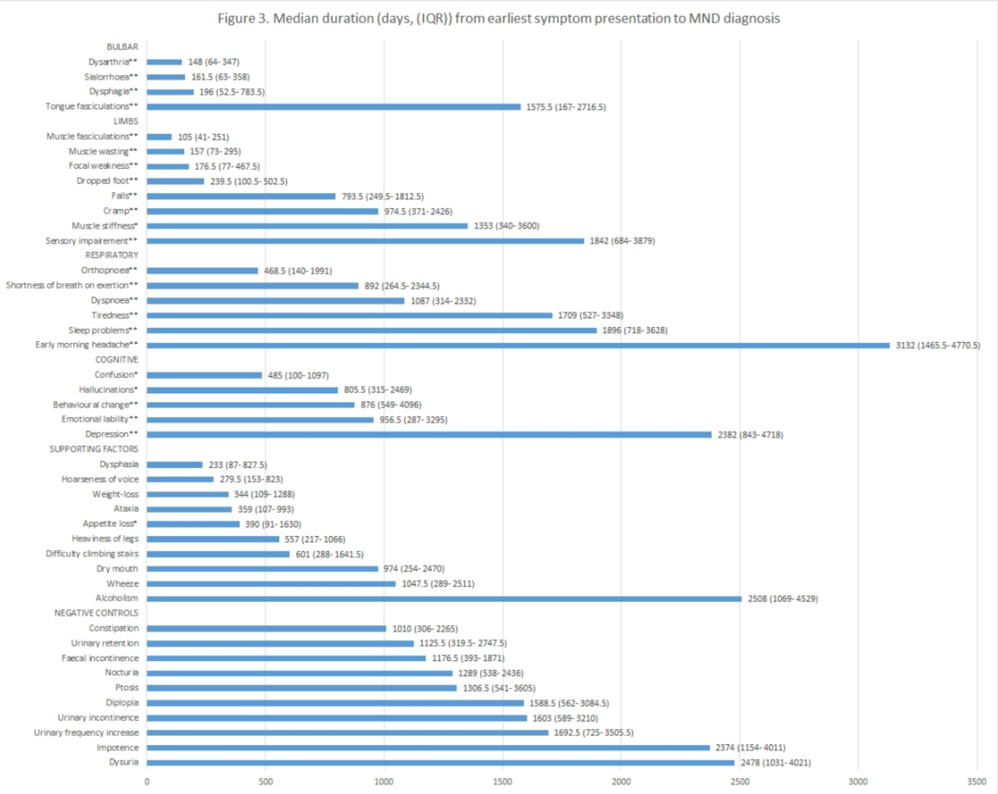

Median duration (days, (IQR)) from earliest symptom presentation to MND diagnosis

562x447mm (57 x 57 DPI)

STROBE Statement—Checklist of items that should be included in reports of ***case-control studies***

	Item No	Recommendation	Page No
**Title and abstract**	1	(*a*) Indicate the study's design with a commonly used term in the title or the abstract	1
		(*b*) Provide in the abstract an informative and balanced summary of what was done and what was found	2
**Introduction**			
Background/rationale	2	Explain the scientific background and rationale for the investigation being reported	3
Objectives	3	State specific objectives, including any prespecified hypotheses	3
**Methods**			
Study design	4	Present key elements of study design early in the paper	3
Setting	5	Describe the setting, locations, and relevant dates, including periods of recruitment, exposure, follow-up, and data collection	3
Participants	6	(*a*) Give the eligibility criteria, and the sources and methods of case ascertainment and control selection. Give the rationale for the choice of cases and controls	3
		(*b*) For matched studies, give matching criteria and the number of controls per case	4
Variables	7	Clearly define all outcomes, exposures, predictors, potential confounders, and effect modifiers. Give diagnostic criteria, if applicable	4
Data sources/ measurement	8*	For each variable of interest, give sources of data and details of methods of assessment (measurement). Describe comparability of assessment methods if there is more than one group	4 for both cases and controls
Bias	9	Describe any efforts to address potential sources of bias	5
Study size	10	Explain how the study size was arrived at	5
Quantitative variables	11	Explain how quantitative variables were handled in the analyses. If applicable, describe which groupings were chosen and why	4
Statistical methods	12	(*a*) Describe all statistical methods, including those used to control for confounding	5
		(*b*) Describe any methods used to examine subgroups and interactions	N/A
		(*c*) Explain how missing data were addressed	5
		(*d*) If applicable, explain how matching of cases and controls was addressed	N/A
		(*e*) Describe any sensitivity analyses	N/A
**Results**			
Participants	13*	(a) Report numbers of individuals at each stage of study—eg numbers potentially eligible, examined for eligibility, confirmed eligible, included in the study, completing follow-up, and analysed	5 for both cases and controls
		(b) Give reasons for non-participation at each stage	N/A
		(c) Consider use of a flow diagram	N/A
Descriptive data	14*	(a) Give characteristics of study participants (eg demographic, clinical, social) and information on exposures and potential confounders	5/6 Table 1 for both cases and controls

For peer review only - http://bmjopen.bmj.com/site/about/guidelines.xhtml

		(b) Indicate number of participants with missing data for each variable of interest	5/6Table 1 for both cases and controls
Outcome data	15*	Report numbers in each exposure category, or summary measures of exposure	9/Figure1 for both cases and controls
Main results	16	(*a*) Give unadjusted estimates and, if applicable, confounder-adjusted estimates and their precision (eg, 95% confidence interval). Make clear which confounders were adjusted for and why they were included	9/Figure1 and 10/Figure 2
		(*b*) Report category boundaries when continuous variables were categorized	N/A
		(*c*) If relevant, consider translating estimates of relative risk into absolute risk for a meaningful time period	N/A
Other analyses	17	Report other analyses done—eg analyses of subgroups and interactions, and sensitivity analyses	N/A
**Discussion**			
Key results	18	Summarise key results with reference to study objectives	10
Limitations	19	Discuss limitations of the study, taking into account sources of potential bias or imprecision. Discuss both direction and magnitude of any potential bias	11
Interpretation	20	Give a cautious overall interpretation of results considering objectives, limitations, multiplicity of analyses, results from similar studies, and other relevant evidence	10/11
Generalisability	21	Discuss the generalisability (external validity) of the study results	N/A
**Other information**			
Funding	22	Give the source of funding and the role of the funders for the present study and, if applicable, for the original study on which the present article is based	12

*Give information separately for cases and controls.

Note: An Explanation and Elaboration article discusses each checklist item and gives methodological background and published examples of transparent reporting. The STROBE checklist is best used in conjunction with this article (freely available on the Web sites of PLoS Medicine at http://www.plosmedicine.org/, Annals of Internal Medicine at http://www.annals.org/, and Epidemiology at http://www.epidem.com/). Information on the STROBE Initiative is available at http://www.strobe-statement.org.

For peer review only

BMJ Open

Identifying Key Signs of Motor Neurone Disease in Primary Care: A Nested Case-Control Study Using the QResearch Database

Journal:	*BMJ Open*
Manuscript ID	bmjopen-2021-058383.R2
Article Type:	Original research
Date Submitted by the Author:	23-May-2022
Complete List of Authors:	Mei, Xue; Oxford University, Nuffield Department of Primary Care Health Sciences<br>Burchardt, Judith; Oxford University, Primary Care Health Sciences<br>Ranger, Tom; Oxford University, Primary Care Health Sciences<br>McDermott, Christopher; The University of Sheffield<br>Radunovic, Aleksandar; Barts Health NHS Trust<br>Coupland, Carol; University of Oxford, Nuffield Department of Primary Care Health Sciences; University of Nottingham<br>Hippisley-Cox, Julia; University of Oxford, Nuffield Department of Primary Care Health Sciences
**Primary Subject Heading**:	Epidemiology
Secondary Subject Heading:	Neurology, Public health, General practice / Family practice
Keywords:	Motor neurone disease < NEUROLOGY, EPIDEMIOLOGY, PRIMARY CARE

SCHOLARONE™
Manuscripts

I, the Submitting Author has the right to grant and does grant on behalf of all authors of the Work (as defined in the below author licence), an exclusive licence and/or a non-exclusive licence for contributions from authors who are: i) UK Crown employees; ii) where BMJ has agreed a CC-BY licence shall apply, and/or iii) in accordance with the terms applicable for US Federal Government officers or employees acting as part of their official duties; on a worldwide, perpetual, irrevocable, royalty-free basis to BMJ Publishing Group Ltd ("BMJ") its licensees and where the relevant Journal is co-owned by BMJ to the co-owners of the Journal, to publish the Work in this journal and any other BMJ products and to exploit all rights, as set out in our licence.

The Submitting Author accepts and understands that any supply made under these terms is made by BMJ to the Submitting Author unless you are acting as an employee on behalf of your employer or a postgraduate student of an affiliated institution which is paying any applicable article publishing charge ("APC") for Open Access articles. Where the Submitting Author wishes to make the Work available on an Open Access basis (and intends to pay the relevant APC), the terms of reuse of such Open Access shall be governed by a Creative Commons licence – details of these licences and which Creative Commons licence will apply to this Work are set out in our licence referred to above.

Other than as permitted in any relevant BMJ Author's Self Archiving Policies, I confirm this Work has not been accepted for publication elsewhere, is not being considered for publication elsewhere and does not duplicate material already published. I confirm all authors consent to publication of this Work and authorise the granting of this licence.

For peer review only

TITLE

Identifying Key Signs of Motor Neurone Disease in Primary Care: A Nested Case-Control Study Using the QResearch Database

Xue W Mei[1], Judith Burchardt[1], Tom A Ranger[1], Christopher J McDermott[2], Aleksandar Radunovic[3], Carol Coupland[1,4], Julia Hippisley-Cox[1]

[1]Nuffield Department of Primary Care Health Sciences, Woodstock Road, University of Oxford

[2]Sheffield Institute for Translational Neuroscience, University of Sheffield, UK

[3]Barts MND Centre, Barts Health NHS Trust, UK

[4] School of Medicine, University of Nottingham, UK

Correspondence to: Professor Julia Hippisley-Cox, Nuffield Department of Primary Care Health Sciences, Radcliffe Observatory Quarter, Woodstock Road, Oxford julia.hippisley-cox@phc.ox.ac.uk @juliahcox

Word count: 3,683 excluding references

For peer review only

ABSTRACT

Objective To confirm the symptoms and signs for MND in the Red Flag tool; to quantify the extent to which the key symptoms and signs are associated with MND; and to identify additional factors which may be helpful within the primary care setting in recognition of possible MND and triggering timely referral to neurology specialists.

Design A nested case-control study.

Setting 1292 UK general practices contributing to the QResearch primary care database, linked to hospital and mortality data.

Participants Baseline cohort included 16.8 million individuals aged 18 years and over without a diagnosis of MND at study entry and with more than 3 years of digitalised information available. The nested case-control dataset comprised of 6,437 cases of MND diagnosed between January 1998 and December 2019, matched by year of birth, gender, general practice, and calendar year to 62,003 controls.

Main outcome measures Clinically recognised symptoms and signs of MND prior to diagnosis and symptoms and factors which are relevant in primary care setting.

Results This study identified 17 signs and symptoms that were independently associated with MND diagnosis in a multivariable analysis. Of these, 7 were new to the Red Flag tool: ataxia, dysphasia, weight-loss, wheeze, hoarseness of voice, urinary incontinence, and constipation. Amongst those from the Red Flag tool, dysarthria had the strongest association with subsequent MND (adjusted odds ratio (AOR): 43.5(95% CI 36.2 to 52.3)) followed by muscle fasciculations (AOR: 40.2 (95% CI 25.6 to 63.1)) and muscle wasting (AOR: 30.6 (95% CI 19.2 to 48.7)). Additionally, the associations between MND diagnosis and family history, dropped foot, focal weakness, and sialorrhoea remained robust after controlling for confounders. Patients that reported symptoms indicative of damage to the lower brainstem and its connections were diagnosed sooner than those who presented with respiratory or cognitive signs.

Conclusion This is the first study that has identified, confirmed, and quantified the association of key symptoms and signs with MND diagnosis. In addition to known factors, the study has identified the following new factors to be independently associated with MND prior to diagnosis: ataxia, dysphasia, wheeze, and hoarseness of voice. These findings may be used to improve risk stratification and earlier detection of MND in primary care.

(363 words)

For peer review only - http://bmjopen.bmj.com/site/about/guidelines.xhtml

STRENGTHS AND LIMITATIONS

- This is the largest nested-case control study derived from a representative population-based cohort looking at signs and symptoms related to MND
- We were able to collate an extensive list of signs and symptoms and to examine the association of each to MND diagnosis
- We were not able to determine the time point, number, and types of specialist referrals
- We do not know about completeness and accuracy of the recording, since not all patients with symptoms will seek medical attention and not all symptoms will be recorded at the GP consultations
- Some observed associations may be due to unmeasured confounding or variations in coding practices at the GPs

INTRODUCTION

Motor Neurone Disease (MND) causes progressive neuromuscular weakness that may first present as isolated and unexplained symptoms. MND causes the deaths of 1 in 350 men and 1 in 470 women in the UK[1], although there is emerging evidence that the incidence might be higher than previously thought[2]. In Europe, the annual crude incidence rate was estimated to be 2.7 per 100,000 person-years[3]. MND is difficult to recognise in primary care since it is both a relatively uncommon disease and the clinical presentations of early symptoms are sporadic and non-specific. As a result, patients may delay consulting their general practitioners (GPs) and the GPs may not attribute the symptoms to MND. Previous work has demonstrated that both patient factors (e.g. delayed presentation to GP) and health care practice factors (e.g. referrals to non-neurology specialists) contribute to delays in MND diagnosis hence patients' access to disease management[4]. The length of diagnostic delay have also been found to be mostly between 10-16 months from symptom onset to diagnosis[4].

In 2014, the Motor Neurone Disease Association and the Royal College of General Practitioners (RCGP) collaborated to produce a Red Flag tool for MND[5], which is designed to improve timely referrals to neurology specialists from primary care consultants. This tool was developed based on interviews with MND specialists and details a number of symptoms and signs which could be indicative of MND but has not been verified by analysis of clinical data. Therefore, the aims of our study are to confirm the symptoms and signs in the Red Flag tool; to quantify the extent to which the key symptoms and signs are associated with MND; as well as to identify additional factors which may be helpful within the primary care setting in recognition of possible MND and triggering timely referral to neurology specialists.

METHOD

Study design

We conducted a nested case-control study in a large UK primary care cohort using the QResearch database (version 44). QResearch is an anonymised, large open cohort database which prospectively collects routine health data from general practices in the UK that use the Egton Medical Information Systems (EMIS) computer system (used by 55% of GPs in the UK). The data include individual level data for demographics, lifestyle factors, medical diagnoses, prescriptions, specialist referrals, medical examinations, and examination results and are linked to Hospital Episode Statistics (HES) records and Office of National Statistics (ONS) death registry records.

For peer review only - http://bmjopen.bmj.com/site/about/guidelines.xhtml

Ascertainment of cases and controls

The base cohort included individuals aged 18 years and over registered during the study period (1 January 1998 to 31 December 2019) without a diagnosis of MND at study entry. The study entry date was defined as the latest of any of the following: 18th birthday; date of registration with the practice plus one year; date on which the practice computer system was installed plus one year; or the beginning of the study period (1 January 1998). The cohort was followed up until the earliest of any of the following: the date of MND diagnosis; date of 100th birthday; date of death; date of leaving the practice; or the study end date (31 December 2019).

Cases were defined as patients in the study cohort with a new diagnosis of MND on any of the GP record, Hospital Episode Statistics (HES) record or Office of National Statistics (ONS) death registry during follow-up. The following Read codes were used to identify the cases from the GP records: F152 (motor neurone disease), F1520 (amyotrophic lateral sclerosis (ALS)), F1521 (progressive muscular atrophy), F1522 (progressive bulbar palsy), F1523 (pseudobulbar palsy), F1524 (primary lateral sclerosis), and F152z (MND not otherwise specified). We also used ICD10 code G122 (motor neurone disease) to identify further records from HES and ONS death registry.

Controls were matched to cases by sex, practice, year of birth, and calendar year using the incidence density sampling method[6]. Each control was allocated an index date which was the date of first MND diagnosis of their matched cases. Each case was matched with up to 10 controls who were alive, registered, and without a diagnosis of MND at the index date. Cases and controls were excluded if they had less than three years of computerised data available at the index date, to ensure that the data are complete for a minimum of three years prior to index date.

Symptoms

Red flag symptoms included clinically recognised symptoms and signs of MND[5 7]. The list of existing factors are as following: bulbar features - dysarthria, dysphagia, sialorrhoea or excessive salivation, and tongue fasciculations; limb or muscle features - focal weakness, falls, foot drop, muscle wasting, muscle fasciculations or twitching, cramps, sensory impairment, and muscle stiffness; respiratory features - orthopnoea, dyspnoea, shortness of breath on exertion, sleep problems, tiredness or fatigue, and early morning headache; cognitive features – behavioural change, emotional lability, depression, hallucinations, and confusion; as well as appetite loss and family history of MND as supporting factors. Additionally, we included potential symptoms such as weight-loss, ataxia, dry mouth, heaviness of legs, and difficulty climbing stairs which were identified through literature searches[8-10] and a search of Read/SNOMED-CT codes for symptoms recorded prior to diagnosis in a pilot study involving a sample of patients diagnosed with MND in JB's general practice. These were included because although not directly established signs/symptoms of MND, these may provide valuable information in identifying additional patients who may benefit from neurologist referral in the primary care setting. We also included the following list of conditions to serve as negative controls since these are not usually considered to be associated with MND: diplopia, ptosis, bladder and bowel problems such as incontinence and constipation, as well as sexual dysfunction. All symptoms and signs were identified through searches of the GP records via corresponding Read/SNOMED-CT codes. For the primary analyses, only signs and symptoms that were last recorded between 3 months to 5 years prior to the index date were included, as symptoms occurring more than 5 years ago were considered unlikely to be relevant to MND and those recorded less than 3 months prior are likely to be reflective of the average waiting period between specialist referral and formal diagnosis. We also excluded very rare signs and symptoms (defined as < 5 ever recorded counts in either cases or

For peer review only - http://bmjopen.bmj.com/site/about/guidelines.xhtml

controls). Table 1 has a summary of the factors included in the analysis and the source of the information.

Statistical analysis

We estimated the strength of association of the red flag signs and symptoms with MND diagnosis, expressed as odds ratios (ORs), using conditional logistic regression. We performed both univariate and multivariable analysis, adjusted for the following confounders: the most recently recorded body mass index (BMI) prior to index date, latest records of smoking status and alcohol consumption, Townsend deprivation score in quintiles, and self-assigned ethnic groups. We assumed missing data on BMI, smoking, alcohol consumption, Townsend scores, and ethnic groups were missing at random and imputed them using multiple imputation by chained equations[11-13]. We created 20 imputed datasets and the imputation model included demographic variables, comorbidities or disease history (I.e. ever diagnosis of one or more of the following: cardiovascular diseases, stroke, diabetes (type 1 and 2), dementia, Parkinson's disease, myasthenia gravis, and multiple sclerosis), all symptoms and signs that were included in the final regression model, as well as the case-control indicator. The ORs from each imputed dataset were combined to form the final adjusted ORs using Rubin's rule[14]. Only signs and symptoms that reached statistical significance (two-tailed $P < 0.05$) in the univariate analysis were included in the multivariable analysis. All statistical analyses were performed using Stata16 (Stata Corp, College Station, TX).

Power calculations

A feasibility study identified over 5,000 patients with MND in the last 20 years in the QResearch. With 5000 cases and 10 matched controls per case we will be able to detect an odds ratio of 1.26 or more for a symptom recorded in 5% of controls, with 90% power and 1% significance. For a symptom recorded in 1% of controls we will be able to detect an odds ratio of 1.59 or more[15].

Patient and public involvement

This study was initiated by the Motor Neurone Disease Association which is a charity representing patients with MND. Apart from this, patients and public were not involved in design and development of the study or interpretation of the study outcome but will be consulted regarding the dissemination of the results.

RESULTS

Descriptive characteristics of the study cohort

The base cohort comprised of 16,799,992 participants aged between 18 and 100 years. During a total of 112,003,453 person-years of follow-up, we found 6,437 incident cases of MND that were eligible for inclusion, using records from general practice, hospital episode statistics, and death registry databases. We were able to identify a total of 62,003 birth year, gender, practice, and calendar year matched controls. 56.8% of the MND cases were men and mean (SD) age at diagnosis was 69.6 (12.2) years.

Table 2 is a summary of the demographic characteristics of the cases and controls. The ethnicity recording was more complete in controls (71.3%) than in cases (55.4%), however, completeness of Townsend deprivation score, alcohol, smoking, and BMI were similar between the two groups. There was no significant difference between cases and controls in terms of alcohol consumption or smoking status. However, compared with the controls, the cases had lower BMI ($P < 0.001$) and were more likely to be living in less deprived areas based on Townsend score quintiles ($P = 0.013$).

For peer review only - http://bmjopen.bmj.com/site/about/guidelines.xhtml

Table 1 Factors included for analysis and the source from which they were identified[1]

	Symptoms and signs	Source/Reason for inclusion	Confirmed on analysis[2]
**Bulbar features**			
	Dysarthria	MNDA	UV, MV
	Dysphagia	MNDA	UV, MV
	Sialorrhoea/excessive salivation	MNDA	UV, MV
	Tongue fasciculations	MNDA	UV
**Limb Features/muscle weakness**			
	Focal weakness	MNDA	UV, MV
	Falls	MNDA	UV, MV
	Foot drop	MNDA	UV, MV
	Muscle wasting	MNDA	UV, MV
	Muscle fasciculations or twitching	MNDA	UV, MV
	Cramps	MNDA	UV, MV
	Sensory impairment	MNDA	No
	Muscle stiffness	NICE	UV, MV
**Respiratory features**			
	Orthopnoea	MNDA	No
	Dyspnoea	MNDA	UV
	Shortness of breath on exertion	MNDA	UV
	Sleep problems	MNDA	UV
	Tiredness/fatigue	MNDA	UV
	Early morning headache	MNDA	No
**Cognitive features**			
	Behavioural change	MNDA	No
	Emotional lability	MNDA	UV
	Depression	MNDA	UV
	Hallucinations	NICE	UV
	Confusion	NICE	UV
**Supporting factors**			
	Family history of MND	MNDA	UV, MV
	Appetite loss	NICE	UV

For peer review only - http://bmjopen.bmj.com/site/about/guidelines.xhtml

**Additional factors from literature review/pilot study**			
	Alcoholism	Literature	No
	Weight-loss	Literature	UV, MV
	Ataxia	Pilot study	UV, MV
	Wheeze	Pilot study	UV, MV
	Difficulty climbing stairs	Pilot study	UV
	Dry mouth	Pilot study	UV
	Dysphasia	Pilot study	UV, MV
	Hoarseness of voice	Pilot study	UV, MV
**Factors not considered supportive of diagnosis by MNDA**			
	Urinary frequency increase	Negative control	UV
	Urinary incontinence	Negative control	UV, MV
	Urinary retention	Negative control	UV
	Nocturia	Negative control	UV
	Dysuria	Negative control	No
	Constipation	Negative control	UV, MV
	Faecal incontinence	Negative control	No
	Impotence	Negative control	No
	Diplopia	Negative control	No
	Ptosis	Negative control	No

[1] The study was matched on age and gender therefore they were not included in the analysis model

[2] Indicates the analysis model used to find the associations. All models were additionally adjusted for Body Mass Index, Townsend scores in quintiles, smoking status, alcohol consumption behaviour, and self-reported ethnicity; UV Univariate, MV Multivariate

Association of red flag signs and symptoms to MND diagnosis

The types of symptoms and signs as well as their adjusted estimates are presented in Figure 1 (univariate) and Figure 2 (multivariate). Table 1 also contains the list of factors examined, source from which they were obtained, and whether they were statistically significant in the analysis. 33 signs and symptoms reached our pre-specified level of statistical significance (two-tailed $P < 0.05$) in the unadjusted univariate analysis, and these remained significant after adjustment for BMI, alcohol consumption, smoking, deprivation, and self-reported ethnicity (Figure 1). These were collectively included in a multivariable model for estimation of their independent associations with MND diagnosis. Dysarthria was the strongest independent predictor of MND diagnosis (OR 43.2, 95%CI 36.0-52.0) followed by muscle fasciculations (OR (95%CI) 40.2 (25.6-63.1)), muscle wasting (OR (95%CI) 31.0 (19.5-49.4)), and dropped foot (OR (95%CI) 14.8 (11.3-19.3)). Some respiratory

For peer review only - http://bmjopen.bmj.com/site/about/guidelines.xhtml

For peer review only

symptoms such as shortness of breath and dyspnoea, as well as cognitive symptoms such as depression and confusion, though statistically significant in univariate analysis, were no longer associated with MND after controlling for other symptoms and signs. We also found the following symptoms, which were not included in the RF tool but were found in GP records, to be independently associated with MND diagnosis: hoarseness of voice (OR (95%CI) 3.26 (2.82-3.77)), dysphasia (OR (95%CI) 2.85 (1.92-4.23)), ataxia (OR (95%CI) 4.82 (3.58-6.48)), wheeze (OR (95%CI) 1.15 (1.01-1.32)), and weight-loss (OR (95%CI) 2.20 (1.92-2.52)).

Table 2. Demographic characteristics of case patients with motor neurone disease and age, sex practice, calendar time matched controls. Figures are numbers (%) unless otherwise indicated.

Characteristic	Study Participants, No. (column %)	
	controls (n = 62,003)	cases (n= 6,437)
Women	27058(43.6)	2781(43.2)
Men	34945(56.4)	3656(56.8)
Age at diagnosis/index date (mean(SD)), y	69.1(11.9)	69.6(12.2)
Age at study entry		
< 50	10700(17.3)	1078(16.7)
50 - 54	6556(10.6)	651(10.1)
55 - 59	8016(12.9)	793(12.3)
60 - 64	9678(15.6)	976(15.2)
65 - 69	9734(15.7)	982(15.3)
70 - 74	8085(13)	852(13.2)
75 - 79	5771(9.3)	627(9.7)
80 +	3463(5.6)	478(7.4)
Ethnicity recorded	44211 (71.3)	3563 (55.4)
**Ethnicity**		
White	41106(66.3)	3281(51)
Indian	700(1.1)	69(1.1)
Pakistani	349(0.6)	32(0.5)
Bangladeshi	174(0.3)	19(0.3)
Other Asian	322(0.5)	30(0.5)
Caribbean	537(0.9)	48(0.7)
Black African	373(0.6)	28(0.4)
Chinese	132(0.2)	4(0.1)
Other	518(0.8)	52(0.8)

For peer review only

For peer review only - http://bmjopen.bmj.com/site/about/guidelines.xhtml

Not recorded	17792(28.7)	2874(44.6)
**Townsend deprivation score, quintiles**		
1 (least deprived)	20410(32.9)	2205(34.3)
2	16131(26)	1613(25.1)
3	11533(18.6)	1174(18.2)
4	8263(13.3)	795(12.4)
5 (most deprived)	5587(9)	640(9.9)
Townsend deprivation score not recorded	79(0.1)	10(0.2)
BMI not recorded before index date	11352(18.3)	1124 (17.5)
BMI (mean(SD))	26.6(4.6)	26.2(4.5)
**Smoking status**		
Non-smoker	29655(47.8)	3142(48.8)
Ex-smoker	17756(28.6)	1882(29.2)
Light smoker (1-9 cigarettes/day)	5931(9.6)	596(9.3)
Moderate smoker (10-19 cigarettes/day)	1125(1.8)	109(1.7)
Heavy smoker (≥20 cigarettes/day)	816(1.3)	76(1.2)
Smoking not recorded before index date	6720(10.8)	632(9.8)
**Alcohol consumption**		
Non-drinker	31677(51.1)	3454(53.7)
Trivial (<1U/day)	9215(14.9)	906(14.1)
Light (1-2u/day)	4460(7.2)	465(7.2)
Moderate (3-6u/day)	4582(7.4)	453(7)
Heavy (7-9u/day)	343(0.6)	30(0.5)
Very Heavy (>9u/day)	129(0.2)	15(0.2)
Alcohol not recorded before index date	11597(18.7)	1114(17.3)

(Figure 1)

We also observed independent associations with MND for two of the symptoms not generally considered to be associated with MND: constipation (OR (95%CI) 1.45 (1.31-1.60)) and urinary incontinence (OR (95%CI) = 1.28 (1.12-1.47)).

Overall, symptoms from bulbar dysfunction (i.e. dysarthria, dysphagia, sialorrhoea, and tongue fasciculations) and those appearing in the limbs are most strongly suggestive of subsequent MND diagnosis. Symptoms and signs occurring in the respiratory and cognitive systems were not statistically significant when evaluated in combination with others.

For peer review only - http://bmjopen.bmj.com/site/about/guidelines.xhtml

For peer review only

(Figure 2)

Time from earliest symptom presentation to MND diagnosis

Duration from the earliest symptom presentation to MND diagnosis varied considerably by feature and location of the signs and symptoms. Patients presenting with symptoms and signs associated with damage of bulbar region and limb functions received their diagnoses sooner than those presenting with respiratory or cognitive symptoms and signs (e.g. median days from symptom presentation to diagnosis: dysarthria 145.5 days (IQR 68.5 – 296 days) vs. orthopnoea 299 days (IQR 42 – 496 days)). Amongst those clinical features, unusual and sporadic signs such as dysarthria and muscle fasciculations appeared to be the key indicators to speedy neurologist referral. Time from earliest clinical symptom presentation to MND diagnosis (median days, IQR) is presented in Figure 3.

(Figure 3)

DISCUSSION

Principal findings

In this very large population based nested case-control study covering a population of over 16 million adults, we were able to demonstrate that the Motor Neurone Disease Association recommended Red Flag signs and symptoms[5], particularly those affecting bulbar and limb regions, are independently predictive of subsequent MND diagnosis. Amongst our study of 6,437 MND patients and 62,003 matched controls, the symptom with the largest association with MND was dysarthria followed by muscle fasciculations and muscle wasting. Additionally from the GP records, we found the following symptoms to be independently associated with MND: hoarseness of voice, dysphasia, ataxia, wheeze, and weight-loss. We also found a higher proportion of MND cases with documented constipation and urinary incontinence than the controls.

Consistent with previous studies[16 17], we found patients showing bulbar onset symptoms such as dysarthria and dysphagia were diagnosed on average 4-5 months sooner than those with limb onset symptoms. Interestingly in our cohort, patients presenting with muscle fasciculations were diagnosed most quickly, which is likely as a result of timely and adequate specialist referral by their managing GPs. This is consistent with a qualitative study conducted by Baxter and McDermott[18] which explored the decision-making and referral process of GPs through semi-structured interviews. The study found that among the 42 GPs who consented to participate, fasciculation was the most commonly described trigger for suspected MND and neurologist referral[18]. Our finding demonstrated that muscle fasciculation is indeed likely one of the top triggers for specialist referral practised by GPs across England. We found the patients who exhibited speech related symptoms such as dysarthria received a diagnosis within an average of 6 months of symptom presentation. This suggests that the awareness of speech impairment in neurological disorders is relatively high amongst GPs in England, in contrast to previous studies[18].

Signs and symptoms of respiratory and cognitive systems, while moderately associated with MND in univariate analysis, were no longer significant after accounting for symptoms related to bulbar region impairment and limb functions. The period between initial presentation of respiratory and cognitive symptoms and MND diagnosis is also significantly longer. This finding seems to indicate that presentations of respiratory or cognitive featured symptoms alone were insufficient to trigger a specialist referral. One reason for this may be that the symptom onset is relatively mild and sporadic,

For peer review only - http://bmjopen.bmj.com/site/about/guidelines.xhtml

and may not alarm the managing GPs since many other patients also have comorbidities with similar symptom profile. However, GPs could cross reference these symptoms against others (i.e. dysarthria or muscle wasting) to enable neurologist referral.

The positive associations between constipation and urinary incontinence with MND found in our study were unexpected since these have not been described in MND before[9 19]. We suspect the observed effect is primarily due to reverse causality: patients with undiagnosed but advanced MND are likely to have trouble with balance and movement, thereby hindering their ability to perform tasks such as going to the toilet.

Strengths and limitations

This study is to date, the largest nested-case control study derived from a representative population-based cohort looking at signs and symptoms related to MND. We were able to collate an extensive list of signs and symptoms from the GP records, and to examine the association of each sign and symptom to subsequent MND diagnosis. We were also able to demonstrate that duration of the earliest symptom presentation to MND diagnosis could serve as an indicator to key signs and symptoms that triggered neurologist referral. We could not determine the time point, number, and types of specialist referrals because our study was designed to compare the pattern of symptoms prior to diagnosis in cases (and the equivalent index date for controls). Another important limitation to our study is the completeness and accuracy of the recording of the signs and symptoms, since not all patients with symptoms will seek medical attention and not all symptoms will be recorded at the GP consultations. Furthermore, the positive associations may be attributed to unmeasured confounding or variation in coding practices at the GPs, due to the nature of routinely collected data. For example, we found dysphasia and wheeze to be independently and positively associated with MND. However these symptoms are not traditionally considered within the clinical features of MND although dysphasia has been described to present in ALS in a systematic review[20]. It might also be that the patients presented with both dysarthria (disorder of speech) and dysphasia (disorder of language) but only dysphasia was coded because it was the dominant symptom when GP consultation was sought. Similarly, shortness of breath on exertion is a well-recognised sign of MND and might be coded as wheeze if symptom presentation of respiratory function tests are not undertaken. The association between ataxia and MND may be due to the fact that “ataxia” is defined according to the clinical code used by GPs to describe what they thought the problem was when they examined the patient. As we now know that these patients subsequently were more likely to be diagnosed with MND, it is likely that what appeared to be “ataxia” or “imbalance due to muscle weakness” when the patient was examined by their GP, may actually have been due to MND.

CONCLUSION

In this very large population based nested case-control study of 6,437 MND cases and 62,003 matched controls, we were able to confirm the currently recognised signs and symptoms, particularly those of bulbar and limb regions to be independently associated with MND. Additionally, we found GP recorded hoarseness of voice, dysphasia, ataxia, and weight-loss to be associated with subsequent MND diagnosis. Due to the nature of the data as highlighted in the limitations, we could not verify whether all positive findings were true symptoms caused by MND or whether they were due to patient presentation patterns or GP coding behaviour. However, these findings have implications for GPs when considering referrals to neurologists and may be used to improve risk stratification and prediction of MND in primary care.

For peer review only - http://bmjopen.bmj.com/site/about/guidelines.xhtml

Future studies may focus on chronologically mapping the symptoms, characterising the patterns and clusters in symptoms presentation, and predicting disease progression based on types and duration of existing symptoms.

CONTRIBUTORS

XM undertook data manipulation, reviewed the literature, led the data analysis, undertook interpretation of the data, and wrote the first draft of the paper. JHC led the study, obtained funding, data approvals, designed the study, drafted the protocol, contributed to the data management and interpretation of the data. CJM contributed to the funding application, contributed to protocol development and interpretation of results. CC contributed to the funding application, the discussion on protocol development and provided critical feedback on drafts of the manuscript. JB reviewed the literature on presentation of MND and conducted a search of how it had presented in patients in her own general practice. TR contributed to interpretation of findings and compilation of the manuscript. XM, JHC, CC, CJM, JB, AR, and TR approved the protocol, contributed to the critical revision of the manuscript and approved the final version of the manuscript. The corresponding author attests that all listed authors meet authorship criteria and that no others meeting the criteria have been omitted.

FUNDING

This study was funded by a grant from Motor Neurone Disease Association. The funders of this study contributed to the design and conduct of the study and reviewed and approve the manuscript. The funders had no role in analysis or interpretation of data. XM, TR, CC, JHC had full access to all the study data and JHC had final responsibility for submission.

COMPETING INTEREST

All authors have completed the ICMJE uniform disclosure form at www.icmje.org/coi_disclosure.pdf. JHC reports grants from grants from the John Fell Oxford University Press Research Fund; Cancer Research UK (CR-UK) grant number C5255/A18085, through the Cancer Research UK Oxford Centre and the Oxford Wellcome Institutional Strategic Support Fund (204826/Z/16/Z), during the conduct of the study. JHC is an unpaid director of QResearch, a not-for-profit organisation which is a partnership between the University of Oxford and EMIS Health who supply the QResearch database used for this work. JHC is a founder and shareholder of ClinRisk ltd and was its medical director until 31st May 2019. JHC is member of the SAGE subgroups on ethnicity and data and is chair of the risk stratification subgroup of NERVTAG. The views expressed are those of the authors only. CJM is supported by the NIHR Sheffield Biomedical Centre.

ETHICAL APPROVAL

The QResearch® ethics approval is with East Midlands-Derby Research Ethics Committee [reference 18/EM/0400, project reference OX1].

DATA SHARING

For peer review only - http://bmjopen.bmj.com/site/about/guidelines.xhtml

To guarantee the confidentiality of personal and health information only the authors have had access to the data during the study in accordance with the relevant licence agreements. Access to the QResearch data is according to the information on the QResearch website (www.qresearch.org).

TRANSPARENCY

XM, TR, and JHC had full access to all data in the study and takes responsibility for the integrity of the data and the accuracy of the data analysis. JHC is the guarantor for the study and affirms that the manuscript is an honest, accurate, and transparent account of the study reported; that no important aspects of the study have been omitted; and that any discrepancies from the study as planned have been explained.

DISSEMINATION TO PARTICIPANTS AND RELATED PATIENT AND PUBLIC COMMUNITIES

PROVENANCE AND PEER REVIEW

Not commissioned; externally peer reviewed.

COPYRIGHT STATEMENT/LICENCE FOR PUBLICATION

"I, the Submitting Author has the right to grant and does grant on behalf of all authors of the Work (as defined in the author licence), an exclusive licence on a worldwide, perpetual, irrevocable, royalty-free basis to BMJ Publishing Group Ltd ("BMJ") its licensees. The Submitting Author accepts and understands that any supply made under these terms is made by BMJ to the Submitting Author unless I am acting as an employee on behalf of my employer which is paying any applicable article publishing charge ("APC") for Open Access articles. Where the Submitting Author wishes to make the Work available on an Open Access basis (and intends to pay the relevant APC), the terms of reuse of such Open Access shall be governed by a Creative Commons licence – details of these licences and which licence will apply to this Work are set out in our licence referred to above."

For peer review only

Figure 1. Univariate analysis for risk of MND diagnosis based on 6,437 cases and 62,003 age, sex, practice matched controls adjusted for variables shown, age, BMI, alcohol, smoking, ethnicity, deprivation

Figure 2. Multivariate analysis for risk of MND diagnosis based on 6,437 cases and 62,003 age, sex, practice matched controls adjusted for variables shown, age, BMI, alcohol, smoking, ethnicity, deprivation

Figure 3. Median duration (days, (IQR)) from earliest symptom presentation to MND diagnosis

For peer review only

For peer review only - http://bmjopen.bmj.com/site/about/guidelines.xhtml

REFERENCES

1. Alonso A, Logroscino G, Jick SS, et al. Incidence and lifetime risk of motor neuron disease in the United Kingdom: a population-based study. *Eur J Neurol* 2009;16(6):745-51.
2. Burchardt JM, Mei XW, Ranger T, et al. Analysis of incidence of motor neuron disease in England 1998–2019: use of three linked datasets. *Amyotrophic Lateral Sclerosis and Frontotemporal Degeneration* 2022:1-9. doi: 10.1080/21678421.2021.2016837
3. Logroscino G, Traynor BJ, Hardiman O, et al. Incidence of amyotrophic lateral sclerosis in Europe. *J Neurol Neurosurg Psychiatry* 2010;81(4):385-90. doi: 10.1136/jnnp.2009.183525 [published Online First: 2009/08/28]
4. Richards D, Morren JA, Pioro EP. Time to diagnosis and factors affecting diagnostic delay in amyotrophic lateral sclerosis. *Journal of the Neurological Sciences* 2020;417:117054. doi: https://doi.org/10.1016/j.jns.2020.117054
5. Association MND. Red Flag Diagnosis Tool [Available from: https://www.mndassociation.org/app/uploads/2013/10/red-flags-final-2.pdf accessed July 2020.
6. Vandenbroucke JP, Pearce N. Case–control studies: basic concepts. *International Journal of Epidemiology* 2012;41(5):1480-89. doi: 10.1093/ije/dys147
7. (NICE) NIfHaCE. Motor neurone disease: assessment and management 2016 [Available from: https://www.nice.org.uk/guidance/ng422019.
8. Kiernan MC, Vucic S, Cheah BC, et al. Amyotrophic lateral sclerosis. *The Lancet* 2011;377(9769):942-55. doi: 10.1016/S0140-6736(10)61156-7
9. McDermott CJ, Shaw PJ. Diagnosis and management of motor neurone disease. *BMJ* 2008;336(7645):658. doi: 10.1136/bmj.39493.511759.BE
10. Nageshwaran S, Davies LM, Rafi I, et al. Motor neurone disease. *BMJ : British Medical Journal* 2014;349
11. Group TAM. Academic Medicine: problems and solutions. *British Medical Journal* 1989;298:573-79.
12. Schafer J, Graham J. Missing data: our view of the state of the art. *Psychological Methods* 2002;7:147-77.
13. Steyerberg EW, van Veen M. Imputation is beneficial for handling missing data in predictive models. *J Epidemiol Community Health* 2007;60:979.
14. Rubin DB. Multiple Imputation for Non-response in Surveys. New York: John Wiley 1987.
15. Dupont WD. Power Calculations for Matched Case-Control Studies. *Biometrics* 1988;44(4):1157-68. doi: 10.2307/2531743
16. Galvin M, Ryan P, Maguire S, et al. The path to specialist multidisciplinary care in amyotrophic lateral sclerosis: A population- based study of consultations, interventions and costs. *PloS one* 2017;12(6):e0179796-e96. doi: 10.1371/journal.pone.0179796
17. Kraemer M, Buerger M, Berlit P. Diagnostic problems and delay of diagnosis in amyotrophic lateral sclerosis. *Clin Neurol Neurosurg* 2010;112(2):103-5. doi: 10.1016/j.clineuro.2009.10.014 [published Online First: 2009/11/26]
18. Baxter S, McDermott CJ. Decision-making and referral processes for patients with motor neurone disease: a qualitative study of GP experiences and evaluation of a new decision-support tool. *BMC Health Serv Res* 2017;17(1):339. doi: 10.1186/s12913-017-2286-0 [published Online First: 2017/05/10]
19. Foster LA, Salajegheh MK. Motor Neuron Disease: Pathophysiology, Diagnosis, and Management. *The American Journal of Medicine* 2019;132(1):32-37. doi: https://doi.org/10.1016/j.amjmed.2018.07.012

For peer review only - http://bmjopen.bmj.com/site/about/guidelines.xhtml

20. Pinto-Grau M, Hardiman O, Pender N. The Study of Language in the Amyotrophic Lateral Sclerosis - Frontotemporal Spectrum Disorder: a Systematic Review of Findings and New Perspectives. *Neuropsychology Review* 2018;28(2):251-68. doi: 10.1007/s11065-018-9375-7

For peer review only

For peer review only - http://bmjopen.bmj.com/site/about/guidelines.xhtml

Figure 1. Univariate analysis for risk of MND diagnosis based on 6,437 cases and 62,003 age, sex, practice matched controls

adjusted for variables shown, age, BMI, alcohol, smoking, ethnicity, deprivation

Symptoms	Cases	Controls	OR (95% CI)	Feature
Dysarthria**	362 (5.6)	124 (0.2)	36.51 (29.27, 45.54)	Bulbar
Sialorrhoea**	29 (0.5)	21 (< 0.1)	12.95 (7.29, 23.03)	Bulbar
Dysphagia**	295 (4.6)	544 (0.9)	6.50 (5.62, 7.52)	Bulbar
Tongue fasciculations**	35 (0.5)	181 (0.3)	1.83 (1.27, 2.65)	Bulbar
Muscle fasciculations**	65 (1)	15 (< 0.1)	50.90 (28.22, 91.80)	Limb
Muscle wasting**	53 (0.8)	21 (< 0.1)	25.69 (15.28, 43.19)	Limb
Dropped foot**	129 (2)	68 (0.1)	20.24 (14.97, 27.37)	Limb
Focal weakness**	364 (5.7)	351 (0.6)	12.19 (10.42, 14.26)	Limb
Falls**	846 (13.1)	3217 (5.2)	3.57 (3.26, 3.92)	Limb
Cramp**	318 (4.9)	1283 (2.1)	2.56 (2.25, 2.91)	Limb
Muscle stiffness*	39 (0.6)	170 (0.3)	2.41 (1.71, 3.41)	Limb
Sensory impairement**	120 (1.9)	1036 (1.7)	1.17 (0.97, 1.42)	Limb
Nocturia	146 (2.3)	841 (1.4)	1.67 (1.39, 2.00)	Respiratory
Shortness of breath**	443 (6.9)	3070 (5)	1.53 (1.37, 1.70)	Respiratory
Tiredness**	470 (7.3)	3251 (5.2)	1.48 (1.33, 1.64)	Respiratory
Dyspnoea**	550 (8.5)	3927 (6.3)	1.47 (1.33, 1.62)	Respiratory
Orthopnoea**	9 (0.1)	66 (0.1)	1.31 (0.65, 2.66)	Respiratory
Sleep problems**	443 (6.9)	3443 (5.6)	1.29 (1.16, 1.44)	Respiratory
Early morning headache**	338 (5.3)	3018 (4.9)	1.11 (0.99, 1.25)	Respiratory
Hallucinations*	13 (0.2)	43 (0.1)	2.55 (1.30, 5.00)	Cognitive
Emotional lability**	26 (0.4)	137 (0.2)	1.95 (1.28, 2.96)	Cognitive
Depression**	263 (4.1)	1521 (2.5)	1.81 (1.57, 2.07)	Cognitive
Confusion*	70 (1.1)	433 (0.7)	1.54 (1.19, 1.99)	Cognitive
Behavioural change**	14 (0.2)	79 (0.1)	1.53 (0.86, 2.74)	Cognitive
Family history of MND**	19 (0.3)	10 (< 0.1)	19.04 (8.80, 41.18)	Supporting factor
Appetite loss*	55 (0.9)	279 (0.4)	1.94 (1.44, 2.61)	Supporting factor
Weightloss	254 (3.9)	910 (1.5)	2.93 (2.53, 3.39)	Literature
Alcoholism	43 (0.7)	357 (0.6)	1.21 (0.88, 1.67)	Literature
Dysphasia	48 (0.7)	52 (0.1)	9.78 (6.49, 14.74)	Pilot study
Ataxia	71 (1.1)	95 (0.2)	7.83 (5.73, 10.71)	Pilot study
Hoarseness of voice	274 (4.3)	518 (0.8)	5.47 (4.69, 6.38)	Pilot study
Difficulty climbing stairs	17 (0.3)	81 (0.1)	2.24 (1.25, 4.00)	Pilot study
Dry mouth	100 (1.6)	575 (0.9)	1.68 (1.35, 2.09)	Pilot study
Wheeze	609 (9.5)	4459 (7.2)	1.46 (1.33, 1.61)	Pilot study
Constipation	628 (9.8)	3119 (5)	2.17 (1.97, 2.38)	Not supporting factor
Urinary incontinence	244 (3.8)	1237 (2)	2.01 (1.74, 2.33)	Not supporting factor
Urinary retention	84 (1.3)	508 (0.8)	1.56 (1.23, 1.98)	Not supporting factor
Ptosis	17 (0.3)	118 (0.2)	1.55 (0.95, 2.53)	Not supporting factor
Faecal incontinence	11 (0.2)	65 (0.1)	1.52 (0.79, 2.91)	Not supporting factor
Diplopia	30 (0.5)	201 (0.3)	1.46 (0.99, 2.14)	Not supporting factor
Urinary frequency increase	214 (3.3)	1515 (2.4)	1.38 (1.19, 1.61)	Not supporting factor
Impotence	251 (3.9)	2378 (3.8)	1.04 (0.91, 1.20)	Not supporting factor
Dysuria	103 (1.6)	984 (1.6)	0.98 (0.80, 1.21)	Not supporting factor

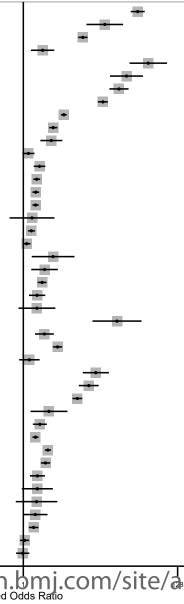

Model included signs and symptoms that were last recorded between 3 months to 5 years prior to MND diagnosis or index date

QResearch database version 44, 2020, University of Oxford, Mei et al

For peer review only - http://bmjopen.bmj.com/site/about/guidelines.xhtml

Figure 2. Multivariate analysis for risk of MND diagnosis based on 6,437 cases and 62,003 age, sex, practice matched controls

adjusted for variables shown, age, BMI, alcohol, smoking, ethnicity, deprivation

Symptoms	Cases	Controls	OR (95% CI)	Feature
Dysarthria**	790 (12.3)	214 (0.3)	43.22 (35.95, 51.97)	Bulbar
Sialorrhoea**	54 (0.8)	35 (0.1)	7.96 (4.50, 14.08)	Bulbar
Dysphagia**	652 (10.1)	1110 (1.8)	4.57 (4.01, 5.21)	Bulbar
Tongue fasciculations**	76 (1.2)	363 (0.6)	1.29 (0.93, 1.78)	Bulbar
Muscle fasciculations**	134 (2.1)	34 (0.1)	40.19 (25.59, 63.13)	Limb
Muscle wasting**	102 (1.6)	39 (0.1)	31.02 (19.46, 49.44)	Limb
Dropped foot**	208 (3.2)	126 (0.2)	14.78 (11.33, 19.28)	Limb
Focal weakness**	736 (11.4)	599 (1)	14.00 (12.20, 16.07)	Limb
Falls**	1304 (20.3)	5290 (8.5)	2.70 (2.46, 2.97)	Limb
Cramp**	470 (7.3)	2427 (3.9)	1.54 (1.35, 1.76)	Limb
Muscle stiffness*	84 (1.3)	446 (0.7)	1.53 (1.15, 2.03)	Limb
Tiredness**	903 (14)	6545 (10.6)	1.08 (0.98, 1.20)	Respiratory
Shortness of breath**	728 (11.3)	5031 (8.1)	1.00 (0.84, 1.18)	Respiratory
Dyspnoea**	819 (12.7)	5990 (9.7)	0.96 (0.84, 1.10)	Respiratory
Sleep problems**	789 (12.3)	6151 (9.9)	0.92 (0.83, 1.02)	Respiratory
Hallucinations*	22 (0.3)	74 (0.1)	1.21 (0.62, 2.35)	Cognitive
Depression**	545 (8.5)	4001 (6.5)	1.05 (0.94, 1.18)	Cognitive
Confusion**	111 (1.7)	613 (1)	0.93 (0.71, 1.21)	Cognitive
Emotional lability**	42 (0.7)	337 (0.5)	0.83 (0.55, 1.24)	Cognitive
Family history of MND**	19 (0.3)	10 (< 0.1)	18.04 (7.64, 42.60)	Supporting factor
Appetitie loss*	109 (1.7)	460 (0.7)	1.26 (0.94, 1.68)	Supporting factor
Weightloss	471 (7.3)	1685 (2.7)	2.20 (1.92, 2.52)	Literature
Ataxia	129 (2)	181 (0.3)	4.82 (3.58, 6.48)	Pilot study
Hoarseness of voice	434 (6.7)	1199 (1.9)	3.26 (2.82, 3.77)	Pilot study
Dysphasia	84 (1.3)	131 (0.2)	2.85 (1.92, 4.23)	Pilot study
Difficulty climbing stairs	24 (0.4)	107 (0.2)	1.46 (0.77, 2.75)	Pilot study
Wheeze	1064 (16.5)	7529 (12.1)	1.15 (1.01, 1.32)	Pilot study
Dry mouth	177 (2.7)	1082 (1.7)	0.97 (0.79, 1.20)	Pilot study
Constipation	958 (14.9)	5253 (8.5)	1.45 (1.31, 1.60)	Not supporting factor
Urinary incontinence	434 (6.7)	2542 (4.1)	1.28 (1.12, 1.47)	Not supporting factor
Nocturia	222 (3.4)	1581 (2.5)	1.08 (0.90, 1.30)	Not supporting factor
Urinary retention	148 (2.3)	942 (1.5)	1.06 (0.85, 1.33)	Not supporting factor
Urinary frequency increase	388 (6)	2982 (4.8)	0.84 (0.73, 0.97)	Not supporting factor

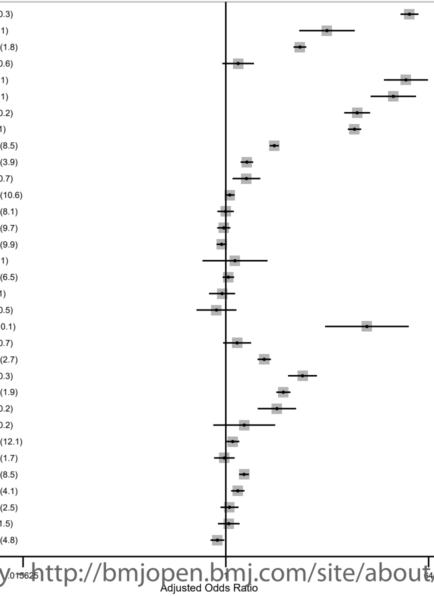

Model included signs and symptoms that were last recorded between 3 months to 5 years prior to MND diagnosis or index date

QResearch database version 44, 2020, University of Oxford, Mei et al

For peer review only - http://bmjopen.bmj.com/site/about/guidelines.xhtml

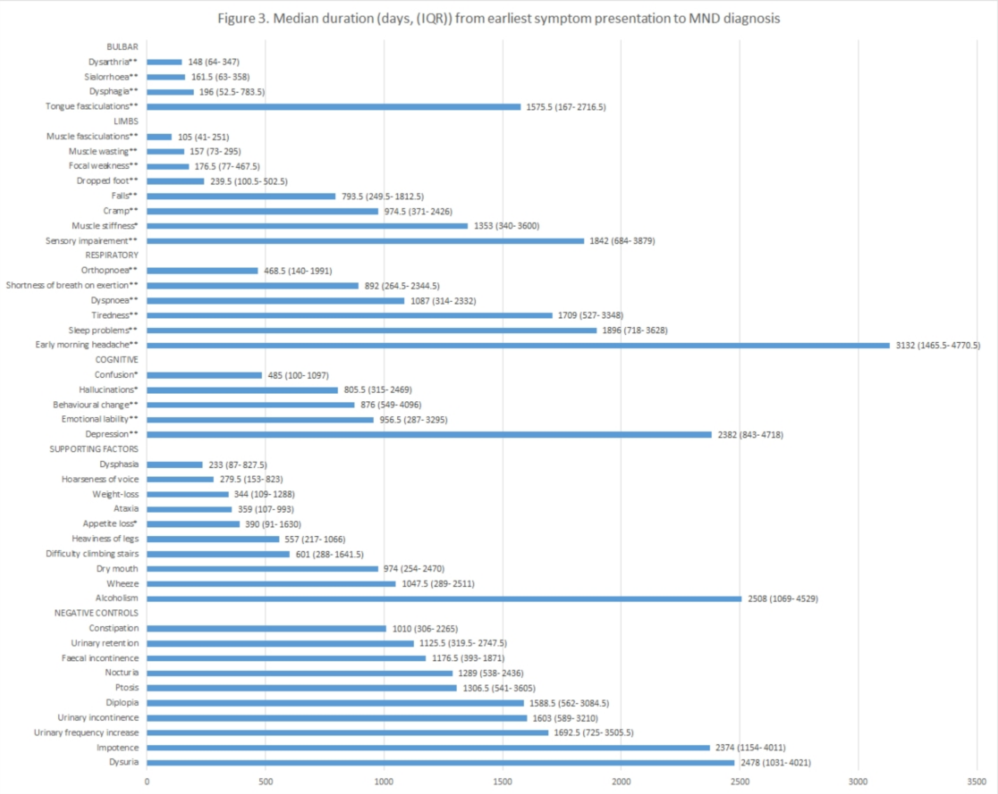

Median duration (days, (IQR)) from earliest symptom presentation to MND diagnosis

562x447mm (57 x 57 DPI)

STROBE Statement—Checklist of items that should be included in reports of ***case-control studies***

	Item No	Recommendation	Page No
**Title and abstract**	1	(*a*) Indicate the study's design with a commonly used term in the title or the abstract	1
		(*b*) Provide in the abstract an informative and balanced summary of what was done and what was found	2
**Introduction**			
Background/rationale	2	Explain the scientific background and rationale for the investigation being reported	3
Objectives	3	State specific objectives, including any prespecified hypotheses	3
**Methods**			
Study design	4	Present key elements of study design early in the paper	3
Setting	5	Describe the setting, locations, and relevant dates, including periods of recruitment, exposure, follow-up, and data collection	3
Participants	6	(*a*) Give the eligibility criteria, and the sources and methods of case ascertainment and control selection. Give the rationale for the choice of cases and controls	3
		(*b*) For matched studies, give matching criteria and the number of controls per case	4
Variables	7	Clearly define all outcomes, exposures, predictors, potential confounders, and effect modifiers. Give diagnostic criteria, if applicable	4
Data sources/ measurement	8*	For each variable of interest, give sources of data and details of methods of assessment (measurement). Describe comparability of assessment methods if there is more than one group	4 for both cases and controls
Bias	9	Describe any efforts to address potential sources of bias	5
Study size	10	Explain how the study size was arrived at	5
Quantitative variables	11	Explain how quantitative variables were handled in the analyses. If applicable, describe which groupings were chosen and why	4
Statistical methods	12	(*a*) Describe all statistical methods, including those used to control for confounding	5
		(*b*) Describe any methods used to examine subgroups and interactions	N/A
		(*c*) Explain how missing data were addressed	5
		(*d*) If applicable, explain how matching of cases and controls was addressed	N/A
		(*e*) Describe any sensitivity analyses	N/A
**Results**			
Participants	13*	(a) Report numbers of individuals at each stage of study—eg numbers potentially eligible, examined for eligibility, confirmed eligible, included in the study, completing follow-up, and analysed	5 for both cases and controls
		(b) Give reasons for non-participation at each stage	N/A
		(c) Consider use of a flow diagram	N/A
Descriptive data	14*	(a) Give characteristics of study participants (eg demographic, clinical, social) and information on exposures and potential confounders	5/6 Table 1 for both cases and controls

For peer review only - http://bmjopen.bmj.com/site/about/guidelines.xhtml

		(b) Indicate number of participants with missing data for each variable of interest	5/6Table 1 for both cases and controls
Outcome data	15*	Report numbers in each exposure category, or summary measures of exposure	9/Figure1 for both cases and controls
Main results	16	(*a*) Give unadjusted estimates and, if applicable, confounder-adjusted estimates and their precision (eg, 95% confidence interval). Make clear which confounders were adjusted for and why they were included	9/Figure1 and 10/Figure 2
		(*b*) Report category boundaries when continuous variables were categorized	N/A
		(*c*) If relevant, consider translating estimates of relative risk into absolute risk for a meaningful time period	N/A
Other analyses	17	Report other analyses done—eg analyses of subgroups and interactions, and sensitivity analyses	N/A
**Discussion**			
Key results	18	Summarise key results with reference to study objectives	10
Limitations	19	Discuss limitations of the study, taking into account sources of potential bias or imprecision. Discuss both direction and magnitude of any potential bias	11
Interpretation	20	Give a cautious overall interpretation of results considering objectives, limitations, multiplicity of analyses, results from similar studies, and other relevant evidence	10/11
Generalisability	21	Discuss the generalisability (external validity) of the study results	N/A
**Other information**			
Funding	22	Give the source of funding and the role of the funders for the present study and, if applicable, for the original study on which the present article is based	12

*Give information separately for cases and controls.

Note: An Explanation and Elaboration article discusses each checklist item and gives methodological background and published examples of transparent reporting. The STROBE checklist is best used in conjunction with this article (freely available on the Web sites of PLoS Medicine at http://www.plosmedicine.org/, Annals of Internal Medicine at http://www.annals.org/, and Epidemiology at http://www.epidem.com/). Information on the STROBE Initiative is available at http://www.strobe-statement.org.

For peer review only
